# Lysosomal pH-inducible supramolecular dissociation of polyrotaxanes possessing acid-labile *N*-triphenylmethyl end groups and their therapeutic potential for Niemann-Pick type C disease

**DOI:** 10.1080/14686996.2016.1200948

**Published:** 2016-07-26

**Authors:** Atsushi Tamura, Kei Nishida, Nobuhiko Yui

**Affiliations:** ^a^ Department of Organic Biomaterials, Institute of Biomaterials and Bioengineering, Tokyo Medical and Dental University, Tokyo, Japan

**Keywords:** Polyrotaxane, cyclodextrin, triphenylmethyl group, cholesterol, Niemann-Pick type C disease, 30 Bio-inspired and biomedical materials, 101 Self-assembly/Self-organized materials, 211 Scaffold/Tissue engineering/Drug delivery

## Abstract

Niemann-Pick type C (NPC) disease is characterized by the accumulation of cholesterol in lysosomes. We have previously reported that biocleavable polyrotaxanes (PRXs) composed of β-cyclodextrins (β-CDs) threaded onto a linear polymer capped with bulky stopper molecules via intracellularly cleavable linkers show remarkable cholesterol reducing effects in NPC disease patient-derived fibroblasts owing to the stimuli-responsive intracellular dissociation of PRXs and subsequent β-CD release from the PRXs. Herein, we describe a series of novel acid-labile 2-(2-hydroxyethoxy)ethyl group-modified PRXs (HEE-PRXs) bearing terminal *N*-triphenylmethyl (*N*-Trt) groups as a cleavable component for the treatment of NPC disease. The *N*-Trt end groups of the HEE-PRXs underwent acidic pH-induced cleavage and led to the dissociation of their supramolecular structure. A kinetic study revealed that the number of HEE groups on the PRX did not affect the cleavage kinetics of the *N*-Trt end groups of the HEE-PRXs. The effect of the number of HEE groups of the HEE-PRXs, which was modified to impart water solubility to the PRXs, on cellular internalization efficiency, lysosomal localization efficiency, and cholesterol reduction ability in NPC disease-derived fibroblasts (NPC1 fibroblasts) was also investigated. The cellular uptake and lysosomal localization efficiency were almost equivalent for HEE-PRXs with different numbers of HEE groups. However, the cholesterol reducing ability of the HEE-PRXs in NPC1 fibroblasts was affected by the number of HEE groups, and HEE-PRXs with a high number of HEE groups were unable to reduce lysosomal cholesterol accumulation. This deficiency is most likely due to the cholesterol-solubilizing ability of HEE-modified β-CDs released from the HEE-PRXs. We conclude that the *N*-Trt group acts as a cleavable component to induce the lysosomal dissociation of HEE-PRXs, and acid-labile HEE-PRXs with an optimal number of HEE groups (4.1 to 5.4 HEE groups per single β-CD threaded onto the PRX) have great therapeutic potential for treating NPC disease.

## Graphical abstract

**Figure:**
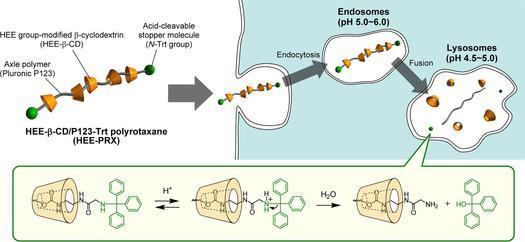

## 1. Introduction

Lysosomes are the primary degradative compartments of cells, and lysosomal enzymes and membrane proteins play a crucial role in the maintenance of cellular homeostasis.[1,2] The materials and proteins internalized via endocytosis or sequestered by autophagy are transferred to lysosomes and degraded by lysosomal enzymes. Lysosomal membrane proteins regulate fusion with other compartments and transportation of the degradation products to other organelles.[1,2] In addition, lysosomal membrane proteins interact with cytoplasmic proteins to modulate intracellular signaling.[3] Because these lysosomal proteins are essential for various cellular functions, congenital deficiencies in these proteins perturb cellular homeostasis. Lysosomal storage disorders are a family of diseases caused by gene mutations in lysosomal enzymes, non-enzymatic proteins, and membrane proteins.[4,5] Lysosomal accumulation of undegraded biochemical metabolites is the hallmark of these diseases. This substrate accumulation underlies the pathogenesis of lysosomal storage disorders, which can cause a variety of serious symptoms, such as neurodegeneration.

Niemann-Pick type C (NPC) disease is a lysosomal storage disorder that is caused by the mutation of either NPC1 or NPC2 proteins.[6–8] Because NPC1 and NPC2 play a central role in the transport of lysosomal cholesterol derived from low-density lipoprotein (LDL) to the endoplasmic reticulum (ER), their deficiencies result in the accumulation of cholesterol in lysosomes.[9,10] As a result of lysosomal cholesterol accumulation, patients with this disease present fatal clinical symptoms such as progressive neurodegeneration and hepatosplenomegaly. Various therapeutic strategies have been proposed for the treatment of NPC disease, such as glucosylceramide synthase inhibitors (i.e. *N*-butyl-deoxynojirimycin),[11,12] histone deacetylase (HDAC) inhibitors (i.e. suberoylanilide hydroxamic acid),[13,14] curcumin,[15] and hydroxypropyl β-cyclodextrins (HP-β-CDs).[16–18] Among these, HP-β-CD has received tremendous attention owing to its strong ability to reduce intracellular cholesterol in NPC1-deficient cells, leading to a prolonged lifespan in mouse and feline models. However, a high concentration of HP-β-CD has recently been reported to exhibit acute toxicity, pulmonary injury, and ototoxicity in a mouse model.[19–23]

**Figure 1.**
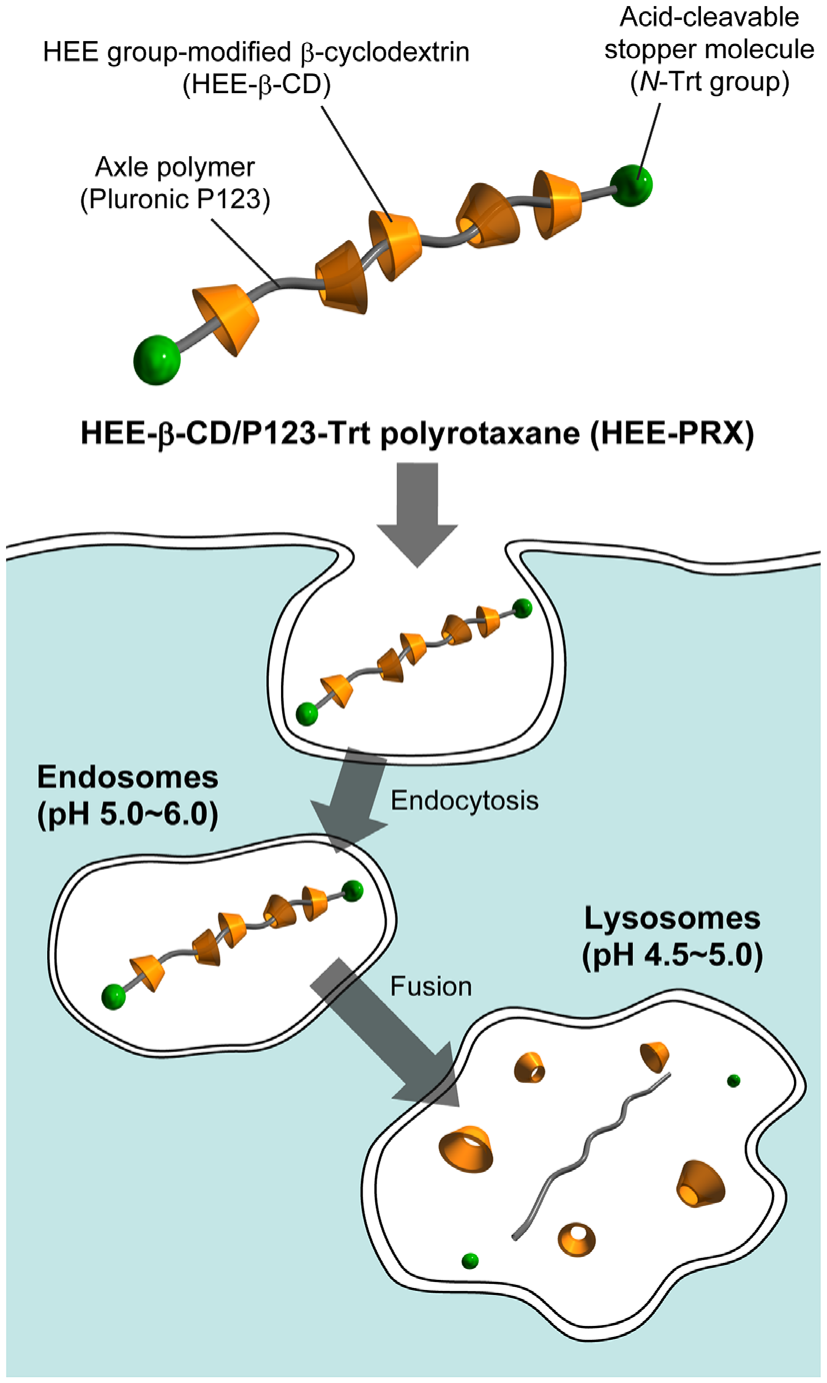
Schematic illustration of HEE group-modified acid-labile polyrotaxanes (HEE-PRXs) and their intracellular dissociation in response to endosomal/lysosomal pH reduction.

One strategy to overcome the toxic issue of β-CD is the use of a polyrotaxane (PRX), a CD-based supermolecule in which the hydrophobic cavity of many CDs is threaded with a linear polymer chain that functions as an intracellular delivery carrier for β-CDs (Figure 1).[24–26] We have previously reported that biocleavable PRXs composed of an axle polymer bearing intracellularly cleavable linkers, threading β-CDs, and bulky stopper molecules are able to dissociate in intracellular environments (Figure 1).[27] Our previous reports demonstrate that the PRX structure masks the toxic effect of β-CDs because the hydrophobic cavity of threaded β-CDs is occupied by a linear polymer chain to perturb the interaction with the plasma membranes (i.e. cholesterol in membranes). As a result, the PRXs can be internalized into cells via endocytosis and undergo intracellular stimuli-induced dissociation to release the threaded β-CDs into the intracellular environment. This local release of abundant β-CDs from the biocleavable PRXs leads to cholesterol reducing ability at a 100-fold lower concentration of clinically studied HP-β-CD and improvements in the impaired autophagy in NPC disease-derived cells.[27,28] Therefore, the biocleavable PRXs are expected to be promising noninvasive and effective therapeutics for the treatment of NPC disease.

From the perspective of materials design, the development of biocleavable PRXs that are responsible for various physical and chemical stimuli and the investigation of their fundamental properties are important issues for expanding therapeutic potential. To date, various cleavable linkers, such as reduction-cleavable disulfide linkages,[27–29] pH-cleavable ester linkages,[30,31] and acid-cleavable hydrazone and ketal linkages,[32–34] have been introduced into the axle polymer of the PRXs to acquire stimuli-dependent dissociative characteristics. Among these, acid-cleavable linkages are of interest to induce lysosomal dissociation of the PRXs because the pH value in lysosomes is maintained at approximately 4.5 to 5.0 by the influx of protons through vacuolar-type H^+^-ATPase (V-ATPase).[35] Although whether the lysosomal pH value remains unaltered in NPC disease is a controversial topic, the pH value in lysosomes has been reported to be acidified (i.e. 4.5 to 5.0) in model cells of NPC disease.[15,36,37] Therefore, acid-labile PRXs are ideal as materials design for lysosomal dissociation and the subsequent release of threaded β-CDs in NPC disease. However, pH-responsive PRXs are considered to be improved in terms of previous deficiencies in stability and synthesis, such as insufficient stability under physiological conditions, dissociation behaviors that occur too slowly under acidic conditions, multi-step synthesis reactions, and a low yield of final product.

Herein, we describe our new findings for biocleavable PRXs possessing an *N*-triphenylmethyl (also called *N*-trityl) (*N*-Trt) end group, which has been used as a stopper molecule for β-CD-threaded PRXs in our previous reports.[27,28,34] It is known that the Trt group is used as a protective group for hydroxyl, thiol, and primary amino groups and is usually deprotected under strongly acidic conditions.[38] However, we found that the *N*-Trt end groups in PRXs were cleaved under mildly acidic pH conditions, such as conditions near lysosomal pH (i.e. pH 4.5 to 5), whereas they exhibited excellent stability under physiological pH conditions. In this study, we synthesized water-soluble 2-(2-hydroxyethoxy)ethyl (HEE) group-modified PRXs bearing *N*-Trt end groups and evaluated their fundamental properties in terms of reaction kinetics for the cleavage of the *N*-Trt end groups at various pH values and temperatures. Additionally, the effect of the number of HEE groups on the PRX on water solubility, cellular internalization efficiency, and cholesterol reducing ability in NPC1-deficient cells was investigated to optimize the chemical parameters of the PRXs for potential use in the treatment of NPC disease.

## 2. Materials and methods

### 2.1. Materials

β-Cyclodextrin (β-CD) was obtained from Nihon Shokuhin Kako (Tokyo, Japan). Pluronic P123, an ABA-type triblock copolymer of poly(ethylene glycol) (PEG) and poly(propylene glycol) (PPG) (PEG-*b*-PPG-*b*-PEG), was obtained from Sigma-Aldrich (Milwaukee, WI, USA) (the number of monomer repeating units in the PEG and PPG segments are 25.2 × 2 (*M*_n,PEG_: 1100 × 2) and 71.5 (*M*_n,PPG_: 4150), respectively). 1,1′-Carbonyldiimidazole (CDI), *N*-triphenylmethyl glycine (Trt-Gly-OH), 2-(2-hydroxyethoxy)ethylamine (HEEA), 2-hydroxypropyl β-cyclodextrin (HP-β-CD) (averaged molecular weight of 1478, the number of HP groups is 5.9 per β-CD), triphenylmethylamine (Trt-NH_2_), dynasore hydrate, and amiloride hydrochloride hydrate were obtained from Sigma-Aldrich. (1*H*-Benzotriazol-1-yloxy)tris(dimethylamino)phosphonium hexafluorophosphate (BOP), ethylenediamine, cholesterol, triphenylmethanol (Trt-OH), and genistein were obtained from Wako Pure Chemical Industries (Osaka, Japan). *N*,*N*-Diisopropylethylamine (DIPEA) was obtained from Tokyo Chemical Industry (Tokyo, Japan). HiLyte Fluor 680 amine (HF680-amine) was obtained from Anaspec (Fremont, CA, USA). The 2-(2-hydroxyethoxy)ethyl group-modified β-cyclodextrins (HEE-β-CDs) were synthesized according to our previous report (see Supplementary text, Figure S1, and Table 1).[28] Other reagents and solvents were obtained from Kanto Chemical (Tokyo, Japan).

**Table 1.** Reaction conditions and characterizations of HEE-PRXs.

Sample code ^a^	Feed [HEEA]/[CDI]/[β-CD] molar ratio ^b^	Number of threading β-CDs ^c^	Number of HEE groups on PRX ^c^	*M*_n,NMR_^d^	*M*_w_/*M*_n_^e^
0.9HEE-PRX	20/2/1	11.1	9.6 (0.9)	22,000	1.07
2.4HEE-PRX	40/4/1	11.1	26.7 (2.4)	24,200	1.08
3.2HEE-PRX	50/5/1	11.1	35.1 (3.2)	25,300	1.09
4.1HEE-PRX	60/6/1	11.1	45.8 (4.1)	26,700	1.08
5.4HEE-PRX	80/8/1	11.1	60.3 (5.4)	28,600	1.09
6.4HEE-PRX	100/10/1	11.1	71.5 (6.4)	30,100	1.09
7.6HEE-PRX	120/12/1	11.1	84.2 (7.6)	31,800	1.09
7.9HEE-PRX	140/14/1	11.1	88.0 (7.9)	32,300	1.09

^a^ Abbreviated as *X*HEE-PRX, where *X* denotes the average number of HEE groups on a single β-CD threaded in the HEE-PRXs.

^b^ The feed molar ratio of [HEEA]/[CDI] was kept constant at 10.

^c^ Determined by ^1^H NMR in DMSO-*d*_6_. The values in parentheses denote the average number of HEE groups on a single β-CD threaded in the HEE-PRXs.

^d^ Calculated based on the chemical composition of the HEE-PRXs determined by ^1^H NMR.

^e^ Determined by SEC in DMSO containing 10 mM LiBr at 60 °C.

### 2.2. Characterization of polyrotaxanes

Size exclusion chromatography (SEC) was performed using a HLC-8120 system (Tosoh, Tokyo, Japan) equipped with a combination of TSKgel α-4000 and α-2500 columns (Tosoh), and eluted with dimethylsulfoxide (DMSO) containing 10 mM LiBr at a flow rate of 0.3 ml min^–1^ at 60 °C. the polydispersity index (*M*_w_/*M*_n_) was calculated from a calibration curve of standard PEGs (Agilent Technologies, Wilmington, DE, USA). ^1^H nuclear magnetic resonance (NMR) spectra were recorded on a Bruker Avance III 500 MHz spectrometer (Bruker BioSpin, Rheinstetten, Germany). Electrospray ionization mass spectrometry (ESI-MS) was performed on a micrOTOF focus II (Bruker Daltonics, Bremen, Germany). ESI-MS spectra were obtained in positive ion electrospray mode.

### 2.3. Synthesis of HEE group-modified PRXs possessing *N*-Trt end groups (HEE-PRXs) and fluorescence-labeled HEE-PRXs

The β-CD/pluronic P123-based polyrotaxanes bearing *N*-Trt end groups were synthesized according to our previous report with slight modifications (see Supplementary text).[28] The typical procedure for the synthesis of 2-(2-hydroxyethoxy)ethyl group-modified PRXs (HEE-PRXs) with various numbers of HEE groups is as follows: PRX (400 mg, 20.4 μmol) and CDI was dissolved in 8 ml of dehydrated DMSO, and the solution was stirred for 24 h at room temperature under a nitrogen atmosphere. Then, HEEA was added to the reaction mixture and the solution was stirred for an additional 24 h at room temperature under a nitrogen atmosphere. After the reaction, the PRX was purified by dialysis against water for three days (Spectra/Por 6, molecular weight cut-off of 3500; Spectrum Laboratories, Rancho Dominguez, CA, USA) at 4 °C. The recovered solution was freeze-dried to obtain HEE-PRX as powder. The detailed reaction conditions are summarized in Table 1. The yield of HEE-PRX was 78–92%. The number of HEE groups that were modified onto the PRX was calculated from the ^1^H NMR peak area between 3.13 ppm (-NH-C***H***_***2***_-CH_2_-O- of HEE group) and 4.7–5.2 ppm (H_1_ proton of β-CD). The *M*_n,NMR_ of HEE-PRX was calculated based on the numbers of threaded β-CDs and HEE groups (Table 1). ^1^H NMR (500 MHz, DMSO-*d*_6_) δ = 1.06 (m, -C***H***_***3***_ of P123), 3.13 ppm (-NH-C***H***_***2***_-CH_2_-O- of HEE group), 3.2–4.3 (m, -C***H***_***2***_C***H***_***2***_O- and -C***H***_***2***_-C***H***- of Pluronic P123 (PEG and PPG), H_2_, H_3_, H_4_, H_5_, and H_6_ protons of β-CD, and -NH-CH_2_-C***H***_***2***_-O-C***H***_***2***_-C***H***_***2***_-OH of HEE group), 4.35–4.7 ppm (m, O_6_H proton of β-CD), 4.7–5.2 (m, H_1_ proton of β-CD), 5.4–6.0 ppm (m, O_2_H and O_3_H protons of β-CD), 7.0–7.15 (m, -N***H***-CH_2_-CH_2_-O- of HEE group), 7.21 (t, Trt group), 7.30 (t, Trt group), 7.42 (d, Trt group).

To synthesize fluorescence-labeled HEE-PRXs, the HEE-PRX (4.1HEE-PRX) (50 mg, 1.87 μmol of HEE-PRX) and CDI (3.37 mg, 20.8 μmol) were dissolved in 1 ml of dehydrated DMSO, and the solution was stirred for 24 h at room temperature under a nitrogen atmosphere. Then, HF680-amine (448 μg, 374 nmol) was added to the reaction mixture, and the solution was stirred for an additional 24 h at room temperature under a nitrogen atmosphere. After the reaction, the PRX was purified by dialysis against water for three days (Spectra/Por 6, molecular weight cut-off of 3500) at 4 °C. The recovered solution was freeze-dried to obtain HF680-modified HEE-PRX (HF680-HEE-PRX) as powder. The number of HF680 modified on PRX was determined by the absorbance at 688 nm, demonstrating that 0.19 HF680 molecules were modified on the HEE-PRX. To adjust the fluorescence intensity among each HF680-HEE-PRX, the HF680-HEE-PRX was mixed with the unlabeled HEE-PRX.

### 2.4. Hydrolysis kinetics for the cleavage of *N*-Trt end groups in HEE-PRXs

The HEE-PRXs were dissolved in buffer solutions at different pH values (pH 4.0 and 5.0: 10 mM CH_3_COOH/CH_3_COONa and 150 mM NaCl, pH 6.0, 7.4, and 8.0: 10 mM NaH_2_PO_4_/Na_2_HPO_4_ and 150 mM NaCl) at a concentration of 5.0 mg ml^–1^, and the solutions were incubated at 37 °C. After incubation for the prescribed time periods, an aliquot of the solutions (100 μl) was collected and mixed with 50 mM NaHCO_3_/Na_2_CO_3_ buffer at pH 9.0 (200 μl) to neutralize the solutions. The solutions were then diluted with acetonitrile (300 μl). The rate of cleavage of *N*-Trt groups from the HEE-PRXs was measured using high performance liquid chromatography (HPLC) setup consisting of an AS-2057i Plus autosampler (Jasco, Tokyo, Japan), a DG-2080–53 degasser (Jasco), a PU-2080i Plus pump (Jasco), a CO-965 column oven (Jasco), an RI-2031 Plus refractive index detector (Jasco), an MD-2018 Plus photodiode array detector (Jasco), and a combination of a Cosmosil 5C_18_-AR-II packed column (250 mm × 4.6 mm internal diameter (ID)) (Nakalai Tesque, Kyoto, Japan) and a Cosmosil 5C_18_-AR-II guard column (10 mm × 4.6 mm ID) (Nakalai Tesque). The solutions (100 μl) were injected into the HPLC system, and the system was eluted with a mixture of water and acetonitrile (the volume ratio of water:acetonitrile was 20:80) at a flow rate of 0.8 ml min^–1^ at 40 °C. The absorption intensities at 205 nm were monitored to determine the rate of cleavage of *N*-Trt groups from the HEE-PRXs. The first-order rate constant (*k*) was determined using the following equation:

$$\ln\left(\frac{{\left[C\right]}_{t}}{{\left[C\right]}_{0}}\right)=-kt$$

where [*C*]_t_ and [*C*]_0_ represent the rate of cleaved *N*-Trt groups at time *t* and 0. The half-life (*t*_1/2_) of the cleavage of *N*-Trt groups in the HEE-PRXs was determined from the following equation:

$$t_{1/2}=\frac{\ln2}{k}$$

To determine the activation energy of the cleavage of *N*-Trt groups in the HEE-PRX, the HEE-PRX solution (5.0 mg ml^–1^, pH 7.4 or 5.0) was incubated at 4, 25, 37, and 50 °C. At the prescribed time periods, aliquots of the solutions were collected, and the cleaved *N*-Trt groups were quantified by HPLC as described above. The activation energy (*E*_a_) for the cleavage of *N*-Trt groups in the HEE-PRX was determined using an Arrhenius plot as follows:

$$\ln k=\ln A-\frac{E_{a}}{RT}$$

where *A* represents the pre-exponential factor, *R* represents the gas constant (8.314 J·K^−1^·mol^−1^), and *T* represents the absolute temperature.

### 2.5. Cell culture

Human skin fibroblasts derived from an NPC disease patient (NPC1) (GM03123) and normal human skin fibroblasts (GM05659) were obtained from the Coriell Institute for Medical Research (Camden, NJ, USA). The cells were cultured in Dulbecco’s modified Eagle’s medium (DMEM) (Wako Pure Chemical Industries) containing 10% fetal bovine serum (FBS) (Sigma-Aldrich), 100 units ml^–1^ penicillin (Wako Pure Chemical Industries), and 100 μg ml^–1^ streptomycin (Wako Pure Chemical Industries) in a humidified 5% CO_2_ atmosphere at 37 °C.

### 2.6. Cellular uptake analysis by flow cytometry and confocal laser scanning microscopy (CLSM) observation

After treatment of normal and NPC1 fibroblasts with the HF680-HEE-PRX (500 μM of β-CD) for 24 h, the cells were washed twice with PBS and harvested by treatment with 0.25% trypsin-ethylenediaminetetraacetic acid (EDTA). Next, the cells were collected by centrifugation (1000 rpm, 4 °C, 5 min) and washed three times with PBS containing 0.1% bovine serum albumin (BSA). After the cells were passed through a 35-μm cell strainer (BD Biosciences, Franklin Lakes, NJ, USA), the fluorescence intensity of the cells was measured using a FACSCanto II system (BD Biosciences). The HF680-HEE-PRXs were excited with a 17-mW He–Ne laser (633 nm) and detected using a 650–670-nm bandpass filter. A total of 10,000 cells were acquired for each sample, and the mean fluorescence intensity of the cell population was analyzed using DIVA software (BD Biosciences). In the inhibition study, the cells were pre-treated with dynasore (100 and 100 μM), genistein (20 and 40 μM), or amiloride (500 and 1000 μM) for 1 h. Then, the cells were treated with HF680-HEE-PRX for an additional 6 h. The fluorescence intensity of the cells was determined as described above.

For the confocal laser scanning microscopy (CLSM) analysis, the cells were treated with the HF680-HEE-PRX (500 μM of β-CD) for 24 h. Next, the cells were stained with LysoTracker Red DND-99 (Thermo Fisher Scientific, Waltham, MA, USA) (100 nM) for 15 min, MitoTracker Red CMXRos (Thermo Fisher Scientific) (100 nM) for 15 min, or ER-Tracker Red (Thermo Fisher Scientific) (2 μM) for 30 min, followed by staining with Hoechst 33342 (Dojindo Laboratories, Kumamoto, Japan) (1 μg ml^–1^) for 10 min at 37 °C. The CLSM observations were performed using a FluoView FV10i (Olympus, Tokyo, Japan) equipped with a 60 × water-immersion objective lens (N/A 1.2).

### 2.7. Filipin staining and cholesterol content

For Filipin staining, the cells were treated with HEE-PRXs for 24 h. Subsequently, the cells were washed twice with PBS and fixed with 4% paraformaldehyde for 15 min at room temperature. The cells were stained with Filipin III (Cayman Chemical, Ann Arbor, Michigan, USA) (50 μg ml^–1^) for 45 min at room temperature. The CLSM observations were performed using a FluoView FV10i.

To determine the content of total cholesterol, the cells were harvested by treatment with 0.25% trypsin-EDTA and lysed with cell lysis buffer (50 mM phosphate buffer, 500 mM NaCl, 25 mM cholic acid, and 0.5% Triton X-100). The total cholesterol content of the cells was determined with an Amplex Red Cholesterol Assay Kit (Molecular Probes). After incubation for 30 min at 37 °C, the fluorescence intensities were measured using an ARVO MX multilabel counter (Perkin Elmer, Wellesley, MA, USA). The total cholesterol content was calculated with a cholesterol standard curve. The protein content in the cell lysate was determined using a Micro BCA Protein Assay Kit (Thermo Fisher Scientific) according to the manufacturer’s instructions. The total cholesterol content of the cells was normalized to the protein content.

### 2.8. Solubility of cholesterol by inclusion complexation with β-CD derivatives

Approximately 2 mg of cholesterol was loaded in a glass vial. Then, 0.5 ml of the aqueous solution of HP-β-CD or HEE-β-CDs (the concentration of HP-β-CD and HEE-β-CDs ranged from 5 to 50 mM) was added to the vial. The suspensions were shaken vigorously for 24 h at 37 °C. Next, the solutions were passed through a 0.22-μm polyvinylidene difluoride (PVDF) membrane filter to eliminate undissolved cholesterol. After the filtrate (300 μl) was diluted with 2-propanol (300 μl), the solubility of the cholesterol was measured with an HPLC setup equipped with a combination of a Cosmosil 5C_18_-AR-II packed column and a Cosmosil 5C_18_-AR-II guard column. The solutions (100 μl) were injected into the HPLC system and the system was eluted with a mixture of 2-propanol and acetonitrile (the volume ratio of 2-propanol:acetonitrile was 40:60) at a flow rate of 1.0 ml min^–1^ at 40 °C.

### 2.9. Statistical analysis

The data are presented as the mean ± standard deviation (SD). Differences between the means of individual groups were assessed by one-way analysis of variance (ANOVA) followed by Tukey-Kramer multiple comparison test. A *p*-value less than 0.05 was considered statistically significant. The statistical analysis was performed using OriginPro 8 (OriginLab, Northampton, MA, USA).

## 3. Results and discussion

### 3.1. Synthesis of water-soluble HEE-PRXs

**Figure 2.**
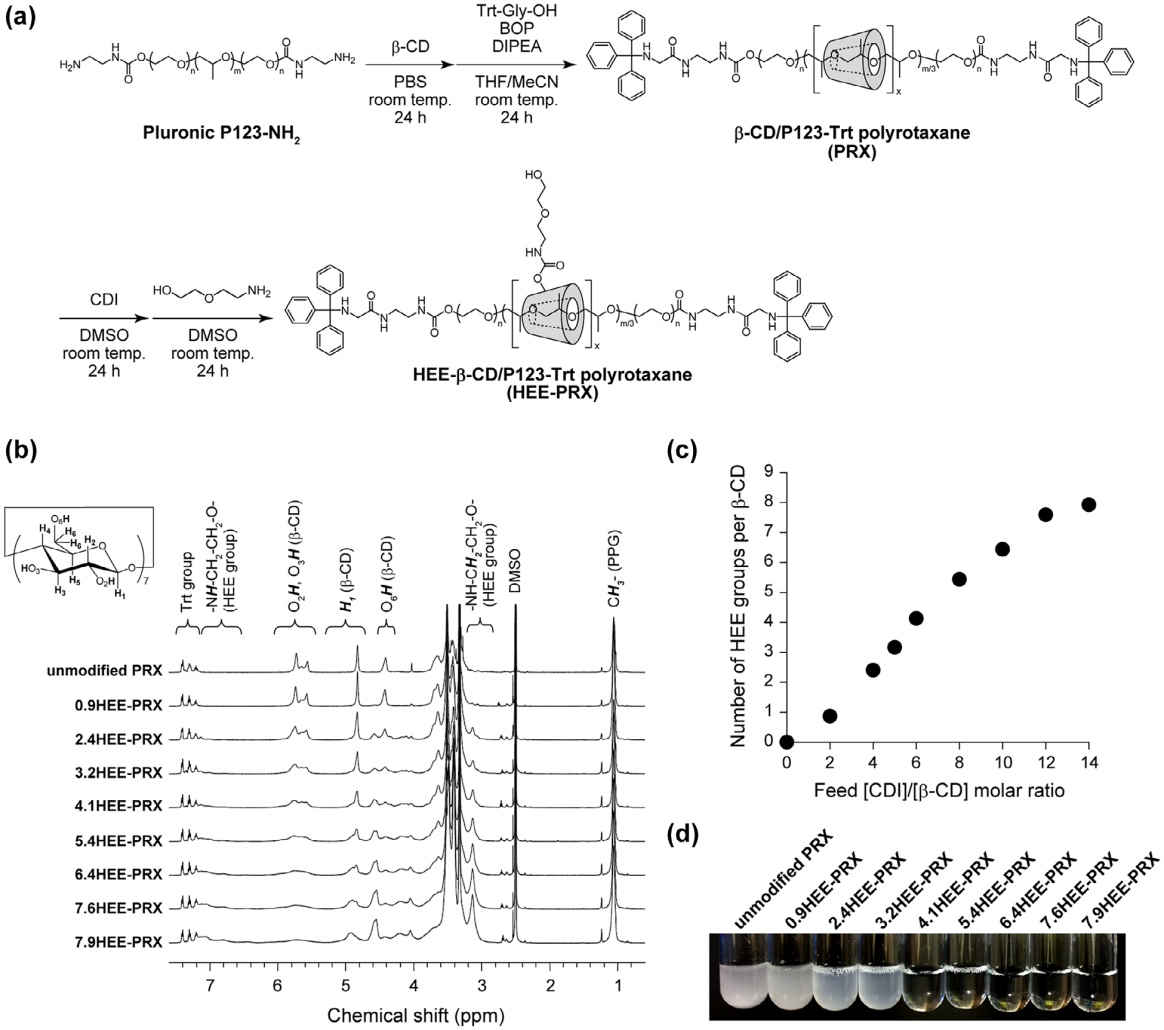
(a) Synthetic scheme of the 2-(2-hydroxyethoxy)ethyl (HEE) group-modified PRXs (HEE-PRXs). (b) ^1^H NMR spectra of the unmodified PRX and the HEE-PRXs (0.9HEE-PRX, 2.4HEE-PRX, 3.2HEE-PRX, 4.1HEE-PRX, 5.4HEE-PRX, 6.4HEE-PRX, 7.6HEE-PRX, and 7.9HEE-PRX) in DMSO-*d*_6_. (c) Relationship between the feed [CDI]/[β-CD] molar ratio and the average number of HEE groups per single β-CD threaded on the HEE-PRXs. (d) Photograph of the unmodified PRX and the HEE-PRXs in water. Concentration of the unmodified PRX and the HEE-PRXs adjusted to 10 mg ml^–1^.

The HEE-PRXs with various numbers of HEE groups were synthesized as shown in Figure 2(a). The pluronic P123 (PEG-*b*-PPG-*b*-PEG triblock copolymer) was selected as an axle polymer, because β-CDs form an inclusion complex with the PPG segment of pluronic P123.[27] In the present study, the β-CD/Pluronic P123-based PRX with 11.1 threading β-CDs was used to synthesize the HEE-PRXs. The number of HEE groups modified on the PRXs varied by the feed [HEEA]/[CDI]/[β-CD in PRX] molar ratios, and the obtained HEE-PRXs were characterized as summarized in Table 1. Note that the SEC charts demonstrate that the HEE-PRXs were successfully synthesized without any impurities such as free β-CDs and HEE group-modified β-CDs (Supplementary Figure S2). In the ^1^H NMR spectra, the HEE groups modified on the PRXs were observed at 3.1 ppm (Figure 2(b)), and the number of HEE groups modified on the threading β-CDs increased almost proportionally with the feed [CDI]/[β-CD in PRX] ratios (Figure 2(c), Table 1). The water solubility of the HEE-PRXs was confirmed by dissolving them in water to a concentration of 10 mg ml^–1^. When the number of HEE groups modified on the threading β-CDs was greater than 4.1, the HEE-PRXs acquired water solubility (Figure 2(d)). The water-soluble HEE-PRXs showed excellent solubility and dissolved in water at concentrations greater than 100 mg ml^–1^, which is significantly higher than previously reported hydroxyethyl group-modified PRXs.[27] In the following experiments, water-soluble 4.1HEE-PRX, 5.4HEE-PRX, 6.4HEE-PRX, and 7.6HEE-PRX were selected to evaluate the effect of the number of HEE groups modified on PRX.

### 3.2. Acid-induced cleavage of *N*-Trt end groups and the dissociation of HEE-PRXs

**Figure 3.**
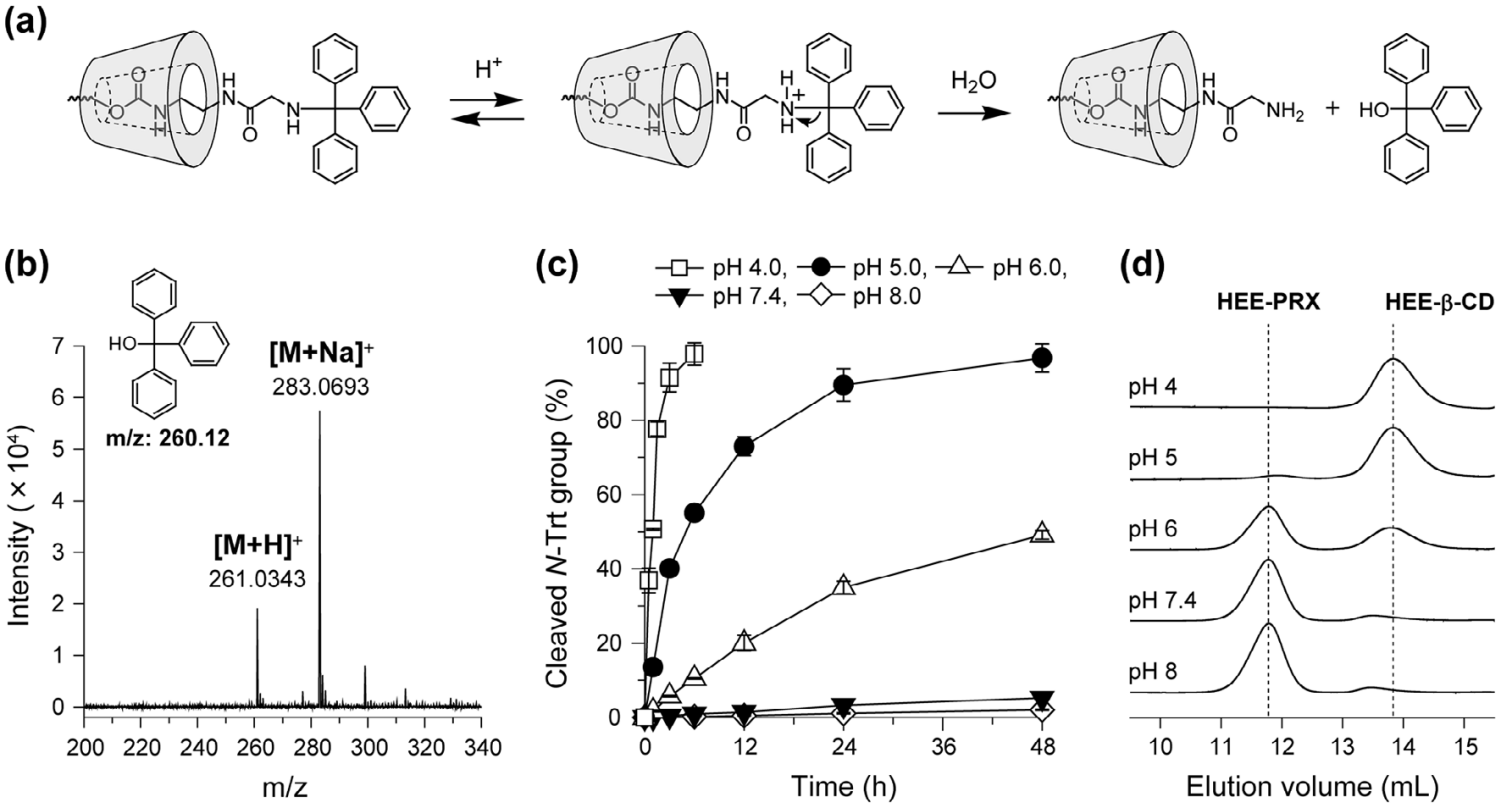
(a) Reaction mechanism for the cleavage of *N*-Trt end groups in HEE-PRX under acidic pH conditions. (b) ESI-MS char of degraded products of HEE-PRX (4.1HEE-PRX) after a 24-h incubation at pH 4.0. (c) Representative time course of the cleavage of *N*-Trt end groups in the HEE-PRX (4.1HEE-PRX) under various pH conditions at 37 °C (open squares: pH 4.0, closed circles: pH 5.0, open triangles: pH 6.0, closed triangles: pH 7.4, and open diamonds: pH 8.0). The data are expressed as the mean ± standard deviation (*n* = 3). (d) Representative SEC charts of HEE-PRX (4.1HEE-PRX) after a 24-h incubation under various pH conditions at 37 °C.

It is known that protonation of the *N*-Trt group under acidic pH conditions results in the liberation of triphenylmethyl cation and ready generation of triphenylmethanol (Trt-OH) via the reaction with water (Figure 3(a)). To confirm the generation of Trt-OH from HEE-PRXs under acidic pH conditions, 4.1HEE-PRX was incubated for 24 h at pH 4.0, and ESI-MS was measured (molar mass of Trt-OH is 260.12). The peaks corresponding to the [Trt-OH + H]^+^ (m/z: 261.0848) and [Trt-OH + Na]^+^ (m/z: 283.0693) were subsequently observed (Figure 3(b)), and other possible peaks such as triphenylmethylamine (Trt-NH_2_) and *N*-triphenylmethyl glycine (Trt-Gly-OH) were not detected (Supplementary Figure S3). This result demonstrated that the *N*-Trt end groups in the HEE-PRXs were cleaved under acidic pH conditions, in accordance with the reaction scheme shown in Figure 3(a).

The effect of pH and time on the cleavage of *N*-Trt end groups in HEE-PRXs was investigated by quantifying the liberated Trt-OH with HPLC. At pHs of 7.4 and 8, cleavage of the *N*-Trt end groups was less than 10% even after 48 h of incubation, indicating that the HEE-PRXs were stable under physiological and alkaline pH conditions (Figure 3(c)). When the pH values decreased, cleavage of the *N*-Trt end groups accelerated. At pH 5.0, which corresponds to the lysosomal pH, the *N*-Trt end groups were completely cleaved within 24 h. Cleavage of the *N*-Trt end groups in HEE-PRXs led to dissociation of the HEE-PRXs. The SEC charts for the HEE-PRXs after incubation for 24 h at various pHs are shown in Figure 3(d). Under acidic pH conditions, the HEE-PRX peaks disappeared, and new peaks derived from unthreaded HEE-β-CDs released from the HEE-PRXs were observed. This result was highly consistent with the cleavage kinetics of the *N*-Trt groups in the HEE-PRXs. Altogether, the HEE-PRXs were sufficiently stable to retain their supramolecular structure under physiological and alkaline pH conditions, whereas under acidic pH conditions, they dissociated to release threaded HEE-β-CDs via cleavage of the *N*-Trt end groups.

**Figure 4.**
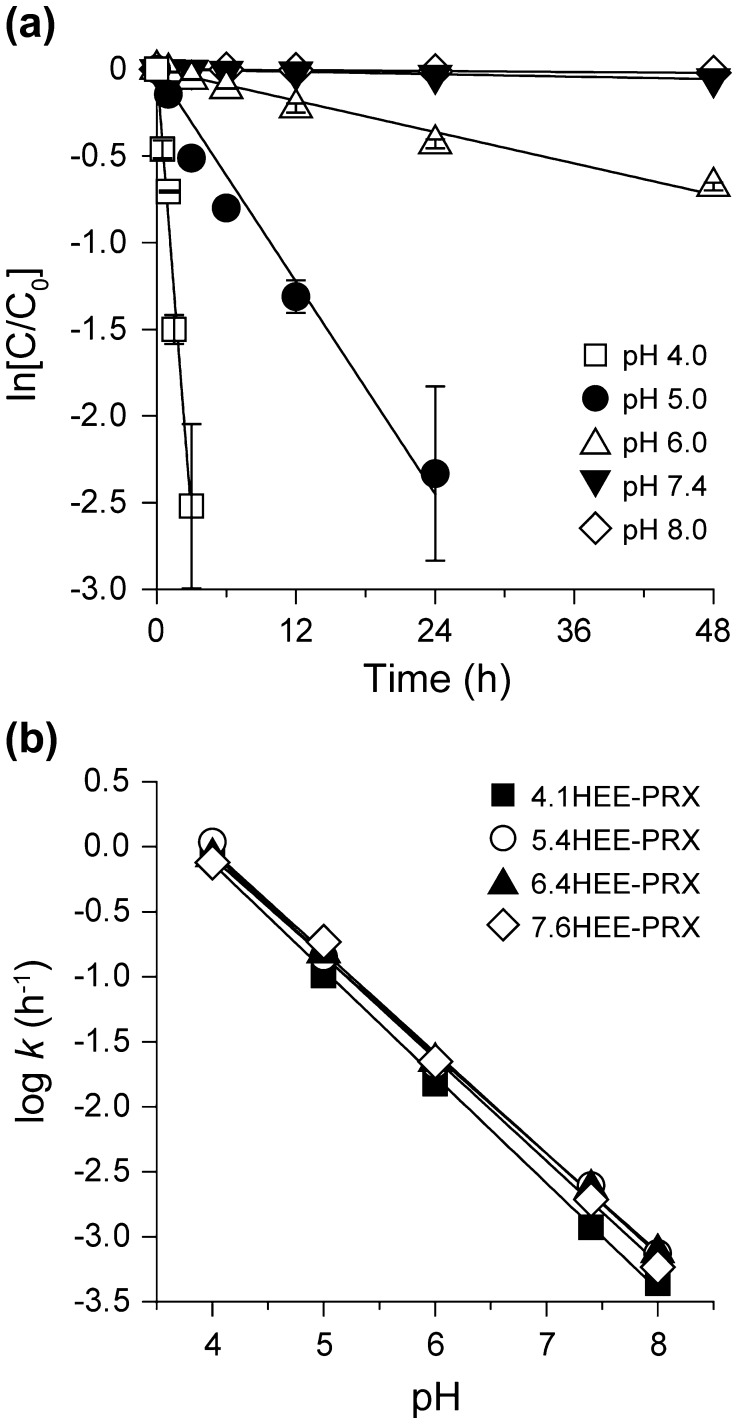
(a) Representative first-order kinetic plots for the cleavage of *N*-Trt end groups in HEE-PRX (4.1HEE-PRX) under various pH conditions at 37 °C (open squares: pH 4.0, closed circles: pH 5.0, open triangles: pH 6.0, closed triangles: pH 7.4, open diamonds: pH 8.0). The data are expressed as the mean ± standard deviation (*n* = 3). (b) Log*k*-pH profiles for the cleavage of *N*-Trt end groups in HEE-PRXs at 37 °C (closed squares: 4.1HEE-PRX, open circles: 5.4HEE-PRX, closed triangles: 6.4HEE-PRX, open diamonds: 7.6HEE-PRX).

To further clarify the acidic cleavage of *N*-Trt group, the kinetics for the cleavage of *N*-Trt end groups in HEE-PRXs was studied. The time course of the ln[C/C_0_] plots for 4.1HEE-PRX revealed that the cleavage of the *N*-Trt end groups was characterized as a first-order reaction under all pH conditions (Figure 4(a)). The same evaluation was also performed for 5.4HEE-PRX, 6.4HEE-PRX, and 7.6HEE-PRX and provided equivalent results (Supplementary Figure S4). The kinetic constant for the cleavage of *N*-Trt end groups in HEE-PRXs under each pH condition was determined based on the slope of the approximate straight lines (Supplementary Table S2). The effect of the number of HEE groups in PRXs on the cleavage kinetics was studied using the log*k*-pH profiles for all HEE-PRXs (Figure 4(b)).[39] As a result, the log*k* values proportionally increased with decreasing pH values, and all HEE-PRXs displayed almost the same log*k*-pH profile. This result suggested that the kinetics for the cleavage of the *N*-Trt end groups in the HEE-PRXs were strongly dependent on the pH (i.e. the proton concentration), and the pH-dependence of the cleavage kinetics was not affected by the number of HEE groups modified on PRXs.

**Figure 5.**
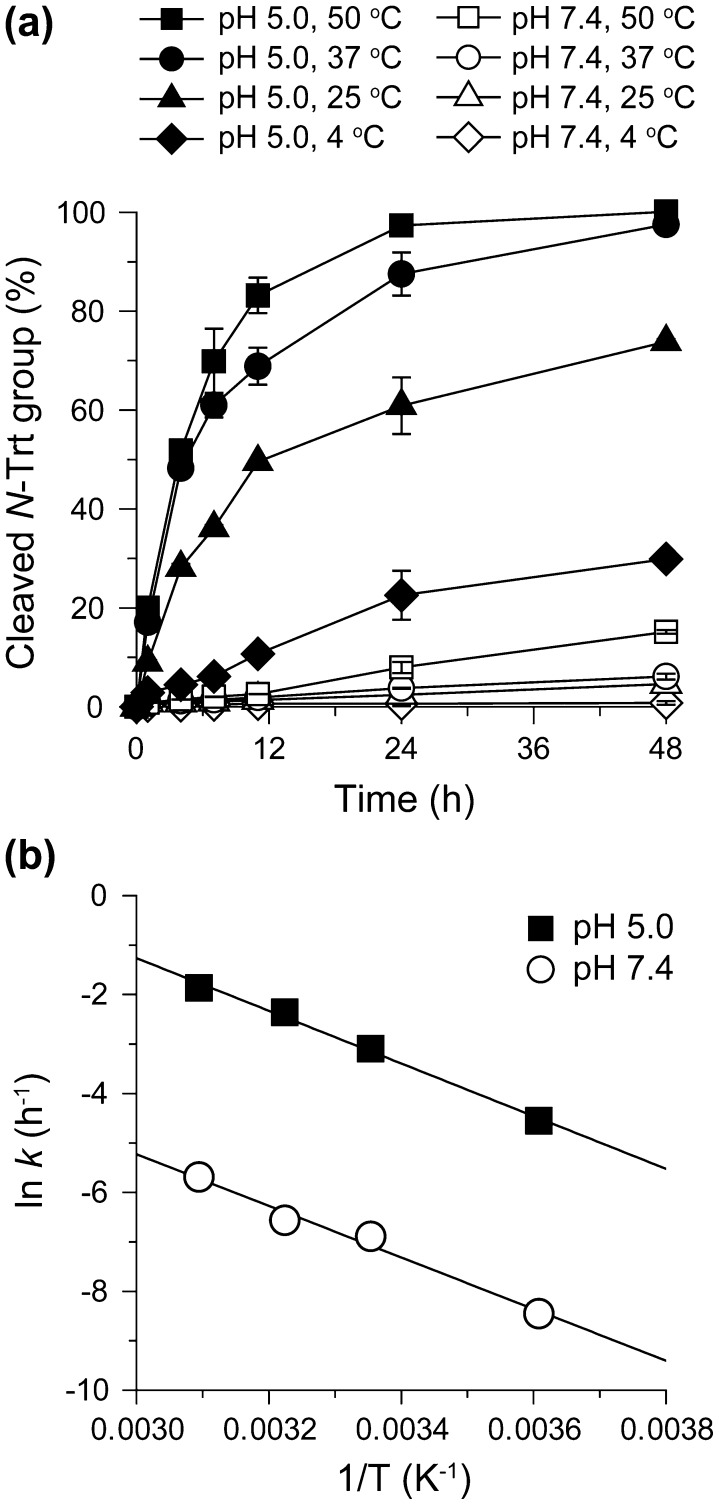
(a) Time course of the cleavage of *N*-Trt end groups in HEE-PRX (4.1HEE-PRX) at pH 5.0 (closed symbols) and 7.4 (open symbols) at various temperatures (squares: 50 °C, circles: 37 °C, triangles: 25 °C, diamonds: 4 °C). (b) Arrhenius plots for the cleavage of *N*-Trt end groups in HEE-PRX (4.1HEE-PRX) at pH 5.0 (closed squares) and 7.4 (open circles).

Temperature is generally one of the important parameters affecting reaction kinetics. To examine the impact of temperature on the cleavage kinetics of *N*-Trt end groups, the cleavage kinetics of 4.1HEE-PRX was assessed at various temperatures under physiological and acidic pHs (pH 7.4 and 5.0) (Figure 5(a)). Under both pH conditions, the cleavage kinetics of the *N*-Trt end groups were clearly accelerated with increasing temperature, and the rate constants differed approximately 10-fold between 4 and 50 °C regardless of the pH (Supplementary Table S3). Figure 5(b) shows the Arrhenius plots for the cleavage of *N*-Trt end groups in 4.1HEE-PRX at pH 7.4 and 5.0. From the results, the activation energies at pH 7.4 and 5.0 were determined to be 44 ± 4 and 47 ± 3 kJ mol^–1^, respectively. The almost equivalent activation energies under different pH conditions suggested that the pre-exponential factor *A* was the major determinant for the cleavage kinetics of the *N*-Trt end groups.

To better understand the stability of the *N*-Trt end groups against various experimental treatments, the stability of the *N*-Trt end groups and the HEE-PRXs against ultrasonication, freeze-thaw cycles, and autoclave treatment was investigated by HPLC and SEC (Supplementary Figure S5). The results revealed that the *N*-Trt end groups were stable against ultrasonication and freeze-thaw cycles, whereas autoclave treatment resulted in cleavage of the *N*-Trt end groups and the dissociation of HEE-PRXs.

### 3.3. Intracellular uptake and cellular localization of HEE-PRXs in normal skin fibroblasts

**Figure 6.**
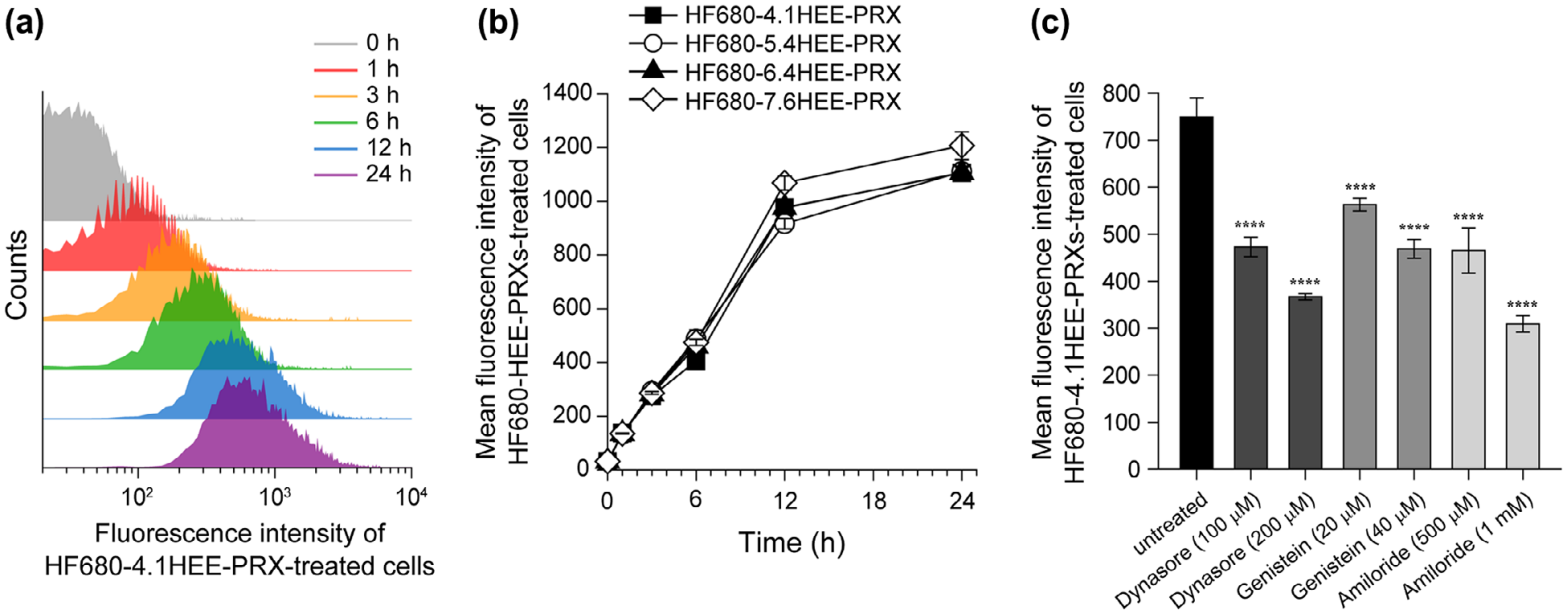
(a) Flow cytometry histograms of normal human skin fibroblasts treated with HF680–4.1HEE-PRX (0.5 mM of β-CD) for various time periods. (b) Time course of the fluorescence intensities of normal human skin fibroblasts treated with HF680-HEE-PRXs (HF680–4.1HEE-PRX: closed squares, HF680–5.4HEE-PRX: open circles, HF680–6.4HEE-PRX: closed triangles, HF680–7.6HEE-PRX: open diamonds) (0.5 mM of β-CD). (c) Effect of endocytosis inhibitors (dynasore for clathrin-mediated endocytosis, genistein for caveolae-mediated endocytosis, and amiloride for macropinocytosis) on the fluorescence intensities of HF680–4.1HEE-PRX-treated normal human skin fibroblasts after 6 h. The data are expressed as the mean ± SD (*n* = 3) (****p < 0.001 vs. untreated cells).

In general, cellular internalization of hydrophilic compounds occurs at a low rate owing to the low partitioning of these compounds into the hydrophobic membrane.[40] Therefore, it is conceivable that the hydrophilic HEE group can disturb cellular internalization of the HEE-PRXs. The effect of the HEE group on cellular internalization of HEE-PRXs was investigated using fluorescence-labeled HEE-PRX (HF680-HEE-PRX). In this experiment, normal human skin fibroblasts were selected as a model cell, and the cellular uptake level (i.e. fluorescence intensity of treated cells) was determined by flow cytometric analysis. When the cells were treated with HF680–4.1HEE-PRX, their fluorescence intensity gradually increased with the treatment duration (Figure 6(a)). Indeed, the fluorescence intensity of the cells increased linearly over 12 h of treatment (Figure 6(b)). In contrast to our expectations, the time course of the cellular fluorescence intensity was almost comparable to the tested HF680-HEE-PRXs with varying numbers of HEE groups. Thus, the number of HEE groups modified on PRX clearly does not affect the cellular internalization efficiency.

The cellular internalization pathway for drugs and materials is an important characteristic in therapeutic applications. Preliminary experiments revealed that cellular uptake of the HEE-PRXs was suppressed at 4 °C, indicating that the cellular internalization pathway of the HEE-PRXs was energy-dependent endocytosis rather than diffusion. Therefore, the type of endocytosis responsible for the cellular internalization of HEE-PRXs was investigated using specific endocytosis inhibitors: dynasore, an inhibitor of dynamin, to examine clathrin-mediated endocytosis,[41] genistein, an inhibitor of several tyrosine kinases, to assess caveolae-mediated endocytosis,[42,43] and amiloride, an inhibitor for Na^+^/H^+^ exchange, to evaluate macropinocytosis [44] (Figure 6(c)). All inhibitors significantly abrogated cellular uptake of HF680-HEE-PRX (HF680–4.1HEE-PRX), indicating that the HEE-PRXs were internalized into cells through various types of endocytosis and were not limited to a specific type of endocytosis.

**Figure 7.**
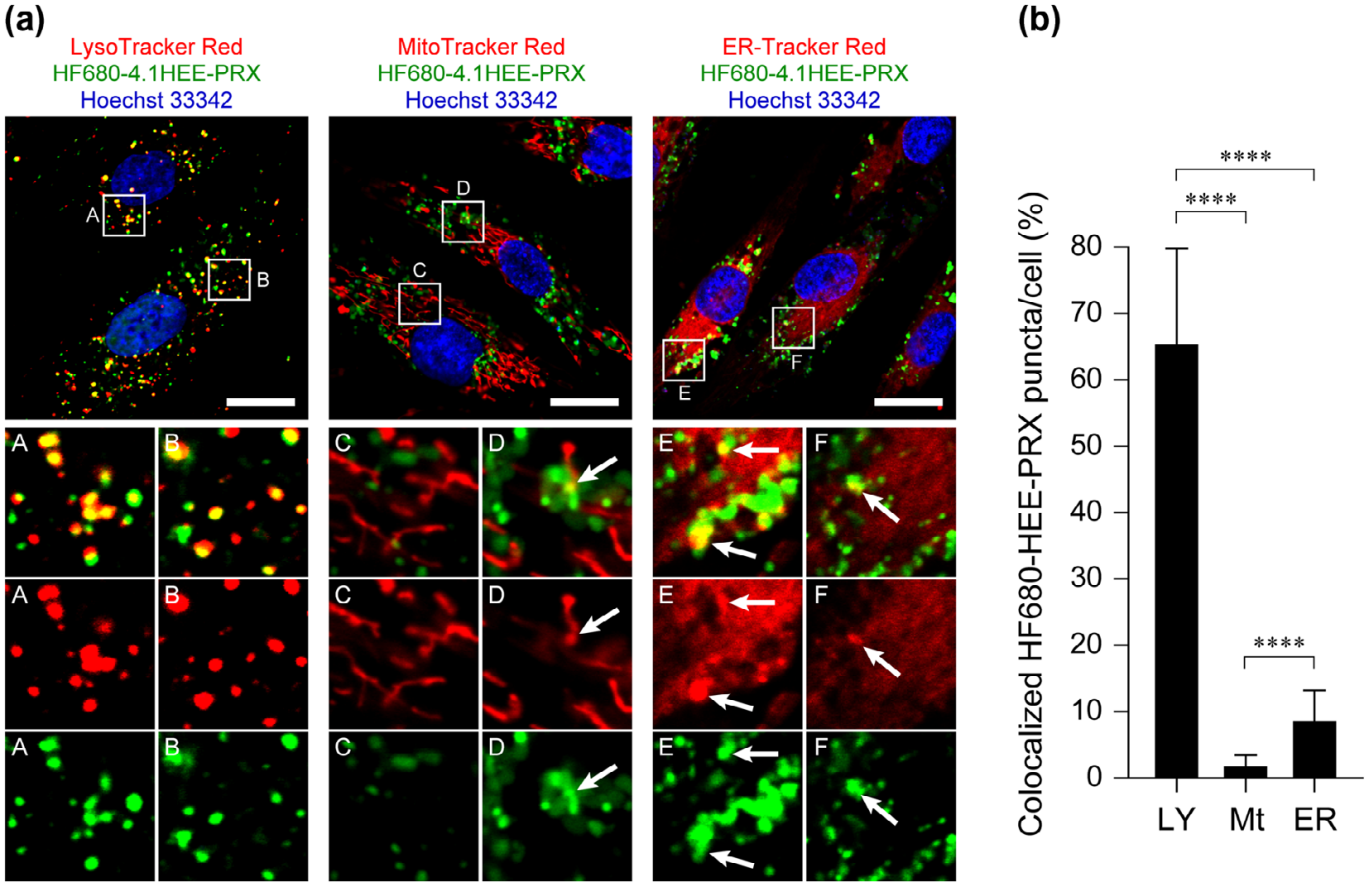
(a) CLSM images of normal human skin fibroblasts treated with HF680–4.1HEE-PRX (0.5 mM of β-CD) for 24 h (scale bars: 20 μm). The endosomes/lysosomes, mitochondria, endoplasmic reticulum, and nuclei were stained with LysoTracker Red DND-99, MitoTracker Red CMXRos, ER-Tracker Red, and Hoechst 33342, respectively. The fluorescence colors are indicated by the color of the text. The bottom panels in each column show enlarged views of the boxed regions. Colocalization of HF680-HEE-PRX with each organelle is indicated by arrows. (b) Percentage of HF680–4.1HEE-PRX-positive puncta that colocalized with endosomes/lysosomes (LY), mitochondria (Mt), and endoplasmic reticulum (ER). The data are expressed as the mean ± SD of 30 cells (****p < 0.001).

The subcellular localization of drugs and materials is another crucial factor for determining therapeutic efficacy. In the case of acid-labile HEE-PRXs, the HEE-PRXs must accumulate in lysosomes and subsequently undergo acid-induced dissociation to release threaded β-CDs for the treatment of NPC disease.[27,28] To investigate the detailed subcellular localization of the HF680-HEE-PRXs (HF680–4.1HEE-PRX) in normal human skin fibroblasts, the major organelles such as lysosomes, mitochondria, and endoplasmic reticulum (ER) were stained with specific indicators and observed by confocal laser scanning microscopy (Figure 7(a)). The results showed that most of the intracellular HF680-HEE-PRXs (shown in green) colocalized with lysosomes (shown in yellow owing to the merge of the green and red signals). In contrast, colocalization of the HF680–4.1HEE-PRXs with mitochondria and ER was rarely observed, although a small amount of colocalization was observed. To further elucidate the localization of HF680–4.1HEE-PRXs, the percentage of HF680–4.1HEE-PRX-positive puncta that colocalized with each organelles was determined by image analysis, as shown in Figure 7(b). The percentages of HF680–4.1HEE-PRX-positive puncta that colocalized with lysosomes, mitochondria and ER were 65, 1.6, and 8.5%, respectively. Although it is difficult to discriminate whether the green signals were derived from HEE-PRXs or the released β-CDs, it is likely that the HEE-PRXs or the released β-CDs preferentially localized in lysosomes.

**Figure 8.**
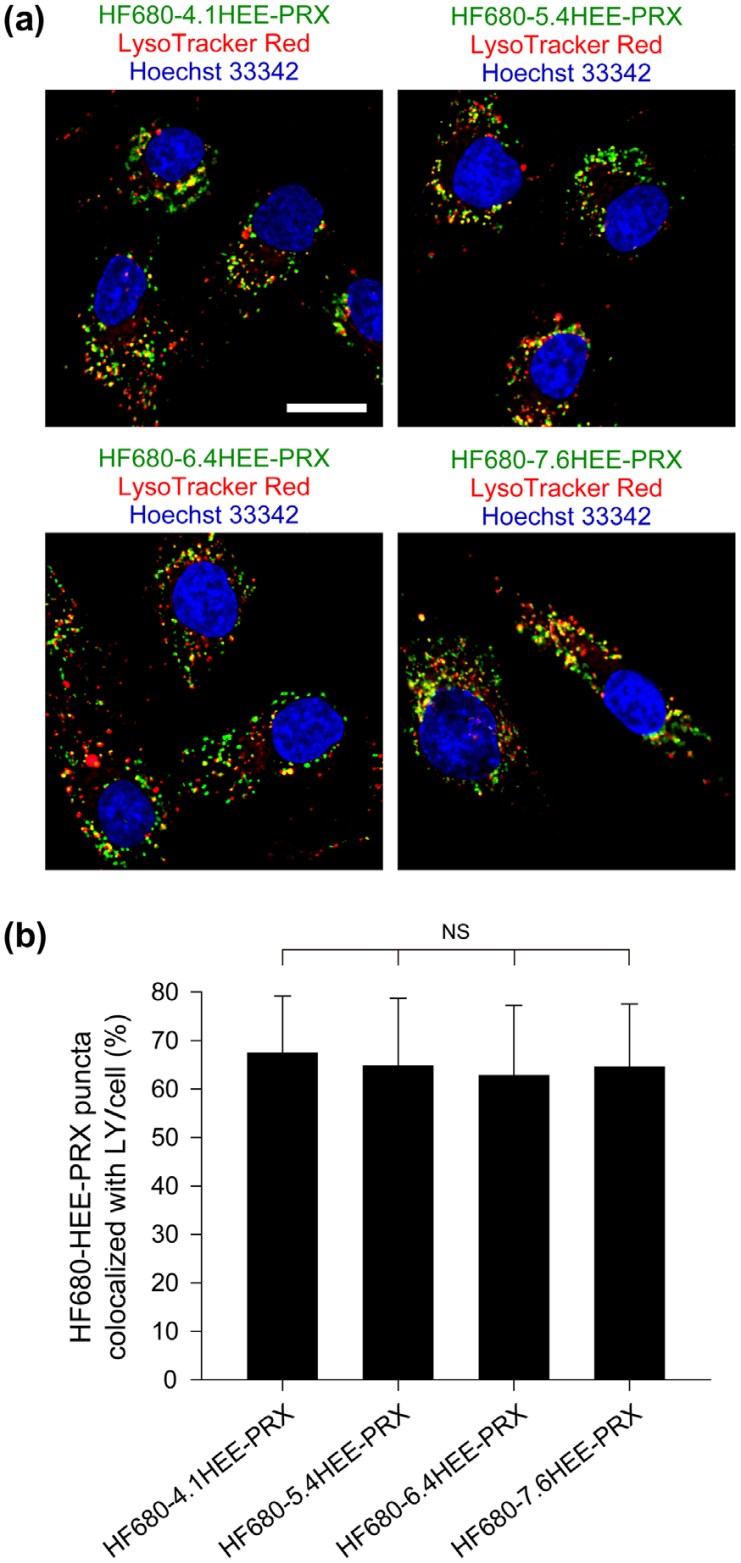
(a) CLSM images of normal human skin fibroblasts treated with HF680-HEE-PRXs (0.5 mM of β-CD) for 24 h (scale bar: 20 μm). The endosomes/lysosomes and nuclei were stained with LysoTracker Red DND-99 and Hoechst 33342, respectively. Fluorescence colors are indicated by the color of the text. (b) Percentage of HF680-HEE-PRX-positive puncta that colocalized with endosomes/lysosomes (LY). The data are expressed as the mean ± SD of 30 cells (NS: not significant).

To confirm whether the preferential lysosomal accumulation of HEE-PRXs was related to the number of HEE groups modified on PRXs, the efficiency of lysosomal localization for four series of HF680-HEE-PRXs was evaluated (Figure 8). Figure 8(a) shows the CLSM images of normal human skin fibroblasts that were treated with HF680-HEE-PRXs followed by staining with LysoTracker Red. Similar to the results shown in Figure 7(a), most of the signals from HF680-HEE-PRXs (shown in green) colocalized with lysosomes (shown in yellow owing to the merge of the green and red signals) for all HF680-HEE-PRXs. The image analysis shown in Figure 8(b) revealed that the lysosomal localization efficiency for four series of HF680-HEE-PRXs was comparable and that were no significant differences (Figure 8(b)). Therefore, the lysosomal localization efficiency of HF680-HEE-PRXs was not affected by the number of HEE groups modified on the PRXs.

### 3.4. Cholesterol reducing effect of HEE-PRXs in NPC disease patient-derived skin fibroblasts

**Figure 9.**
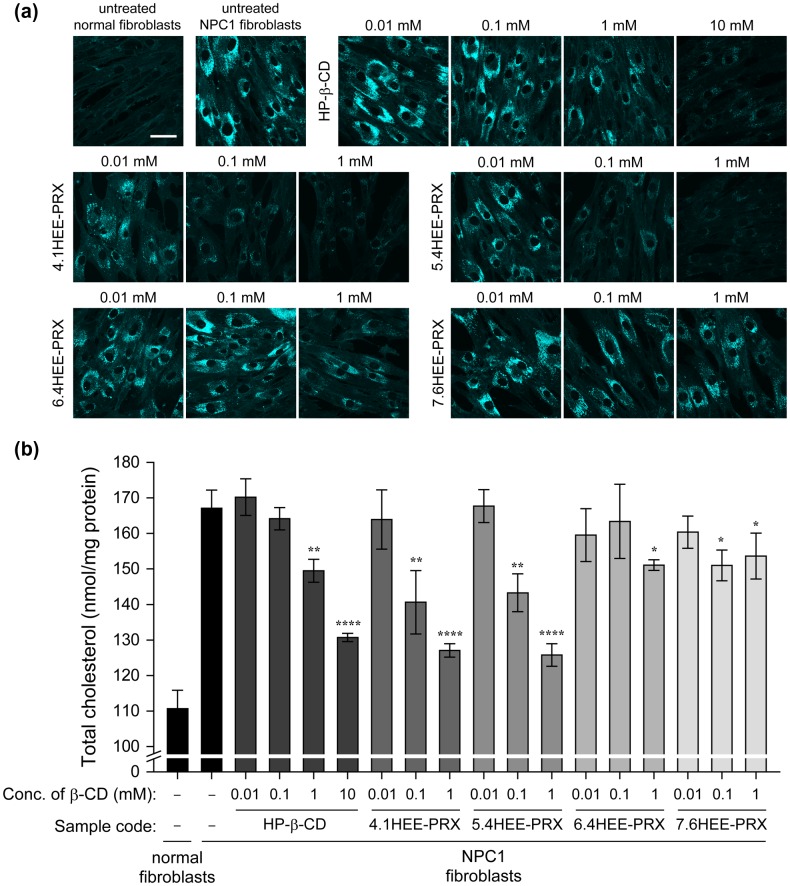
(a) Filipin-stained normal and NPC1 fibroblasts treated with HP-β-CD (0.01, 0.1, 1 and 10 mM) and HEE-PRXs (0.01, 0.1, and 1 mM of β-CD) for 24 h (scale bars: 50 μm). (b) The amount of total cholesterol in normal and NPC1 fibroblasts treated with HP-β-CD (0.01, 0.1, 1, and 10 mM) and the HEE-PRXs (0.01, 0.1, and 1 mM of β-CD) for 24 h. The data are expressed as the mean ± SD (*n* = 3) (*p < 0.05, **p < 0.01, ****p < 0.001 vs. untreated NPC1 fibroblasts).

To evaluate the effect of the number of HEE groups modified on PRX for the treatment of NPC disease, the intracellular cholesterol content in NPC disease patient-derived skin fibroblasts (NPC1 fibroblasts) was investigated by Filipin staining, a cholesterol binding fluorescence molecule,[45] and quantification of the intracellular total cholesterol using an enzymatic method [46] (Figure 9). In this experiment, HP-β-CD was used as a control because it is expected to have a promising therapeutic effect in NPC disease.[16–18] Figure 9(a) shows the Filipin staining of normal and NPC1 fibroblasts after treatment with HP-β-CD and HEE-PRXs (4.1HEE-PRX, 5.4HEE-PRX, 6.4HEE-PRX, and 7.6HEE-PRX) for 24 h. NPC1 fibroblasts clearly exhibited higher Filipin fluorescence signals than normal fibroblasts. The fluorescence signals of Filipin gradually decreased to normal levels with an increasing concentration of HP-β-CD, 4.1 HEE-PRX, and 5.4HEE-PRX, whereas the fluorescence signals of Filipin remained unchanged following treatment with 6.4HEE-PRX and 7.6HEE-PRX. Quantification of the intracellular total cholesterol content resulted in similar tendencies: HP-β-CD, 4.1HEE-PRX, and 5.4HEE-PRX could reduce the intracellular cholesterol level in a concentration-dependent manner, whereas 6.4HEE-PRX and 7.6HEE-PRX could not reduce the cholesterol content (Figure 9(b)). It is noteworthy that 4.1 HEE-PRX and 5.4HEE-PRX could reduce lysosomal cholesterol at a lower concentration than HP-β-CD, which is consistent with previous results.[27,28,34] As mentioned above, the *N*-Trt end groups of HEE-PRXs should be cleaved in acidic lysosomes and subsequently release threaded β-CDs. Therefore, special interest should be paid to the limited cholesterol reducing effect of HEE-PRXs by the number of HEE groups on PRX.

It is unclear why HEE-PRXs with a high number of HEE groups (6.4HEE-PRX and 7.6HEE-PRX) weakened the cholesterol reducing ability in NPC1 fibroblasts because the acid-induced cleavage kinetics of the *N*-Trt groups in each HEE-PRX (Figure 4(b)) and the cellular uptake level of each HEE-PRX in NPC1 fibroblasts were almost equivalent for all HEE-PRXs (Supplementary Figure S6). Additionally, HEE-PRX was preferentially accumulated in lysosomes of NPC1 fibroblasts, similar to the results obtained for normal fibroblasts (Supplementary Figure S7). Ours and other groups have previously reported that methylated β-CD has superior cholesterol reducing ability compared with HP-β-CD, because methylated β-CD has higher ability to form an inclusion complex with cholesterol than HP-β-CD. A possible explanation for this phenomenon could be a difference in ability to form an inclusion complex between cholesterol and the β-CDs released from HEE-PRXs.[27]

**Figure 10.**
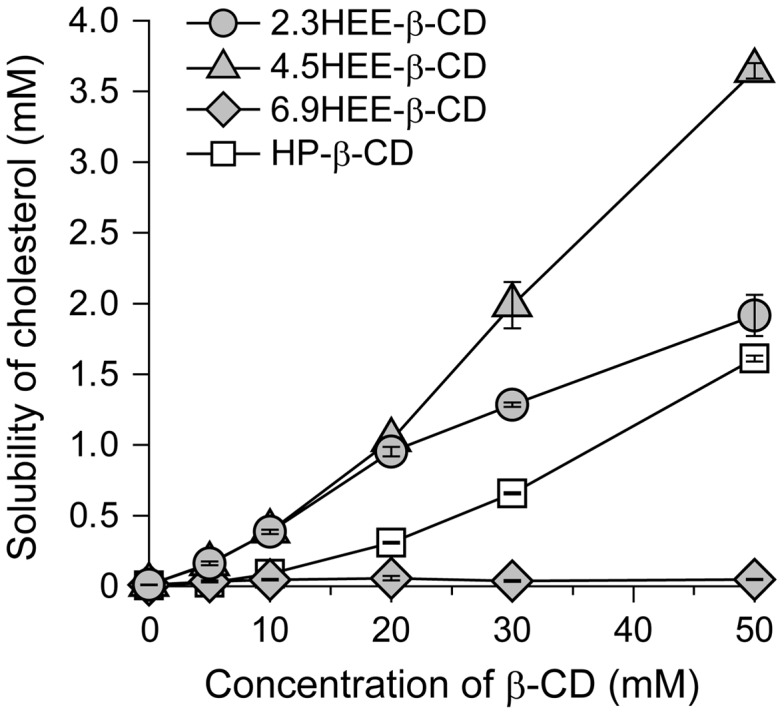
Phase-solubility diagrams of cholesterol with 2.3HEE-β-CD (filled circles), 4.5HEE-β-CD (filled triangles), 6.9HEE-β-CD (filled diamonds), and HP-β-CD (open squares) in PBS. The solubility of cholesterol was determined after incubation for 24 h at 37 °C (*n* = 3).

To validate this hypothesis, HEE group-modified β-CDs (HEE-β-CD) with various numbers of HEE groups were synthesized (Supplementary Table S1), and their ability to form an inclusion complex with cholesterol was investigated by measuring the solubility of cholesterol (Figure 10). The number of HEE groups in the HEE-β-CDs was not identical to that in the HEE-PRXs because precise regulation of the number of HEE groups in HEE-β-CDs was difficult. The results of a phase-solubility study demonstrated that inclusion complexation between cholesterol and HEE-β-CDs depended strongly on the number of modified HEE groups, and highly HEE group-modified HEE-β-CD (6.9HEE-β-CD) lost the ability to form an inclusion complex with cholesterol. Thus, a high number of HEE groups on 6.9HEE-β-CD is considered to hinder inclusion complexation with cholesterol owing to a lack of intermolecular interactions or steric repulsion between cholesterol and 6.9HEE-β-CD. The cholesterol solubilizing ability of the optimal HEE-β-CD (4.5HEE-β-CD) was characterized as an A_P_-type diagram (A-type with a positive deviation from linearity) similar to HP-β-CD, indicating that more than one HEE-β-CD molecule is involved in the complexation with cholesterol.[47,48] A similar result has been reported for the inclusion complex system between HP-β-CD and digitoxin.[49] It is noteworthy that the 4.5 HEE-β-CD exhibited significantly higher cholesterol solubility than HP-β-CD. Additionally, the cholesterol solubilizing ability of 2.3HEE-β-CD was higher than HP-β-CD, especially at low concentrations, but it appeared to be characterized as an A_N_-type diagram (A-type with a negative deviation from linearity).[46, 47] Consequently, the effect of the number of HEE groups on HEE-PRXs on cholesterol reduction in NPC1 fibroblasts was due to the inclusion complexation ability of the unthreaded HEE-β-CDs released from HEE-PRXs rather than the kinetics for the intracellular dissociation, cellular uptake level, and lysosomal accumulation efficiency of HEE-PRXs.

In our previous study, we confirmed that the interlocked PRX structure masks the toxic effect of β-CD derivatives, because the interlocked structure inhibits exposure of the toxic hydrophobic cavity of β-CDs.[27] The HEE-PRXs described in this study also showed negligible toxicity in normal human skin fibroblasts even at the threaded β-CD concentration of 30 mM (Supplementary Figure S8). In contrast, the viability of normal human skin fibroblasts decreased following treatment with 20 mM of HP-β-CD owing to exposure of the hydrophobic cavity (Supplementary Figure S8). Altogether, the acid-labile HEE-PRXs with an optimal number of HEE groups (4.1 to 5.4 HEE groups per single β-CD threaded onto the PRX) have great potential to improve lysosomal cholesterol accumulation in NPC disease without marked toxicity.

## 4. Conclusions

In summary, a series of acid-labile HEE-PRXs bearing terminal *N*-Trt groups as a cleavable component were developed as a therapeutic approach to NPC disease. Although *N*-Trt groups are usually used as protective groups in organic chemistry, the present study demonstrated their feasibility as an acid-responsive element in biomaterials science and pharmacology. As shown herein, the *N*-Trt group is an ideal component for achieving the stability of PRXs under physiological conditions as well as for inducing lysosomal their pH-induced dissociation. We conclude that the *N*-Trt group is promising as a cleavable component for inducing lysosomal dissociation of HEE-PRXs. Additionally, acid-labile HEE-PRXs with an optimal number of HEE groups (4.1 to 5.4 HEE groups per single β-CD threaded onto the PRX) possess great therapeutic potential for treating NPC disease. It will be extremely important to evaluate the safety and therapeutic efficacy of acid-labile HEE-PRXs in a mouse model of NPC disease.

## Funding

This work was supported by the Grant-in-Aid for Scientific Research on Innovative Areas ‘Nanomedicine Molecular Science’ from the Ministry of Education, Culture, Sports, Science, and Technology (MEXT) of Japan [number 23107004 to NY]; Grant-in-Aid for Young Scientists (B) from Japan Society for the Promotion of Science (JSPS) [number 26750155 to AT]; Grant-in-Aid for Young Scientists (A) from JSPS [number 16H05910 to AT]; Adaptable and Seamless Technology Transfer Program through Target-driven R&D (A-STEP) from Japan Science and Technology Agency (JST) [number AS262Z01189Q to AT]; The Mochida Memorial Foundation for Medical and Pharmaceutical Research [to AT]; The Sasakawa Scientific Research Grant from The Japan Science Society [to AT]; Azuma Medical & Dental Research Grant [to AT].

## Disclosure statement

No potential conflict of interest was reported by the authors.

## Supplemental data

Supplemental data for this article can be accessed here. http://dx.doi.org/10.1080/14686996.2016.1200948

## Supplementary Material

160720_corrected_supplementary_file.pdf
