# Supplementary material for: Lysosomal pH-inducible supramolecular dissociation of polyrotaxanes possessing acid-labile N-triphenylmethyl end groups and their therapeutic potential for Niemann-Pick type C disease
Source: Sci Technol Adv Mater. 2016 Jul 26;17(1):361–74. doi: 10.1080/14686996.2016.1200948 (PMC5101866; doi:10.1080/14686996.2016.1200948)
Supplement: 160720_corrected_supplementary_file.pdf [file tsta_a_1200948_sm3945.pdf]

Supplementary data

Lysosomal pH-inducible supramolecular dissociation of polyrotaxanes possessing acid-labile *N*-triphenylmethyl end groups and their therapeutic potential for Niemann-Pick type C disease

Atsushi Tamura, Kei Nishida, and Nobuhiko Yui

Department of Organic Biomaterials, Institute of Biomaterials and Bioengineering,

Tokyo Medical and Dental University, 2-3-10 Kanda-Surugadai, Chiyoda, Tokyo 101-0062, Japan.

To whom correspondence should be addressed: N.Y. (E-mail: yui.org@tmd.ac.jp)

Synthesis of β-CD/Pluronic P123-based polyrotaxanes bearing *N*-Trt end groups

Pluronic P123 (30.0 g, 4.71 mmol) and CDI (11.5 g, 70.6 mmol) were loaded into a round-bottomed flask and dissolved in 270 mL of dehydrated tetrahydrofuran (THF). The solution was stirred for 24 h at room temperature under a nitrogen atmosphere. Next, the reaction mixture was added dropwise to the dehydrated THF solution (100 mL) of ethylenediamine (15.8 mL, 235.5 mmol). The solution was stirred for 24 h at room temperature under a nitrogen atmosphere. After the reaction, the polymer was purified by dialysis against methanol for 3 days (Spectra/Por 6, molecular weight cut-off of 1,000 Da). Finally, the solvent was removed under reduced pressure to obtain α,ω-bisamino Pluronic P123 (P123-NH_2) (16.8 g, 54.5% yield). ^{1}H NMR (500 MHz, $CDCl_3$) δ = 1.16 (m, 214H, C$\boldsymbol{H_3}$ of PPG), 3.40 (m, 71H, -CH_2-C***H-*** of PPG), 3.54 (m, 142H, -C$\boldsymbol{H_2}$-CH- of PPG), 3.64 (m, 202H, -C$\boldsymbol{H_2}$-C$\boldsymbol{H_2}$-O- of PEG), 4.35 (t, 4H, -C$\boldsymbol{H_2}$-O(=O)-NH-).

A saturated solution of β-CD was prepared by dissolving β-CD (80.0 g, 70.5 mmol) in 4 L of phosphate buffer saline (PBS) (10 mM NaH_2PO_4/Na_2HPO_4, 150 mM NaCl). Next, P123-SS-NH_2 (7.0 g, 1.07 mmol) dissolved in a small aliquot of water was added to the β-CD saturated solution, and the mixture was stirred for 24 h at room temperature, during which a precipitate of pseudopolyrotaxane was obtained. After the reaction, the precipitate was collected by centrifugation (7,000 rpm, 3 min) and freeze-dried for 1 day to obtain pseudopolyrotaxane as powder (41.5 g). The Trt-Gly-OH (10.1 g, 32.0 mmol), BOP (14.1 g, 32.0 mmol), and DIPEA (5.5 mL, 32.0 mmol) were then dissolved in the mixture of THF and acetonitrile (THF:acetonitrile at a 1:1 volume ratio), and the solution was added to the pseudopolyrotaxane. The resulting reaction mixture was stirred for 24 h at room temperature. After the reaction, the precipitate was collected by centrifugation (7,000 rpm, 3 min). The precipitate was dissolved in DMF, and the solution was poured into water to form precipitate. The precipitate was collected by centrifugation (7,000 rpm, 3 min). The reprecipitation process was repeated six times to completely remove the free β-CD and unreacted reagents. Finally, the precipitate was washed with water to remove the DMF. The recovered precipitate was freeze-dried to obtain PRX (3.02 g, 14.5% yield based on Pluronic P123 mol%). The number of β-CDs threaded onto the P123 was determined from the ^{1}H NMR peak area between 4.84 ppm (H_1 proton of β-CD) and 1.06 ppm (-C$\boldsymbol{H_3}$ of Pluronic P123). ^{1}H NMR (500 MHz, DMSO-d_6) δ = 1.06 (m, -C$\boldsymbol{H_3}$ of P123), 3.1-3.9 (m, -C$\boldsymbol{H_2}$C$\boldsymbol{H_2}$O- and -C$\boldsymbol{H_2}$-C$\boldsymbol{H}$- of Pluronic P123 (PEG and PPG), H_2, H_3, H_4, H_5, and H_6 protons of β-CD), 4.42 ppm (m, O_6H proton of β-CD), 4.83 ppm (m, H_1 proton of β-CD), 5.5-5.9 ppm (m, O_2H and O_3H protons of β-CD), 7.21 (t, Trt group), 7.30 (t, Trt group), 7.42 (d, Trt group).

Synthesis of 2-(2-hydroxyethoxy)ethyl group-modified β-CDs (HEE-β-CDs)

To a solution of β-CD (1.0 g, 881 μmol) in anhydrous DMSO (15 mL), CDI was added at room temperature. After the reaction for 24 h, HEEA was added to the reaction mixture and stirred for an additional 24 h at room temperature. The PRX was then purified by dialysis against methanol for 4

days (Spectra/Por 6, molecular weight cut-off of 1,000 Da). The recovered solution was evaporated and dissolved in water. The aqueous solution was freeze-dried to obtain HEE-β-CD. The number of modified HEE groups on β-CD was determined by ^{1}H NMR. ^{1}H NMR (500 MHz, D_2O) δ = 3.24 (m, -C$\boldsymbol{H_2}$$CH_2$-O-$CH_2CH_2$-OH of HEE group), 3.5-4.5 (m, H_2, H_3, H_4, H_5, and H_6 protons of β-CD, -CH_2C$\boldsymbol{H_2}$-O-C$\boldsymbol{H_2}$C$\boldsymbol{H_2}$-OH of HEE group), 4.95 ppm (m, H_1 proton of β-CD).

Cytotoxicity assay

Normal human skin fibroblasts were plated in a 96-well plate at a density of 1×10^4 cells/well and incubated overnight. After the medium was replaced with fresh medium (90 μL), the treatment solutions (10 μL) were added to each well. After incubation for an addtional 24 h, Cell Counting Kit-8 reagent (Dojindo Laboratories, Kumamoto, Japan) (10 μL) was added to each well. After further incubation for 1.5 h at 37 °C, the absorbance at 450 nm was measured using a Multiskan FC plate reader (Thermo Fisher Scientific, Waltham, Massachusetts, USA). The cellular viability was calculated relative to the untreated cells.

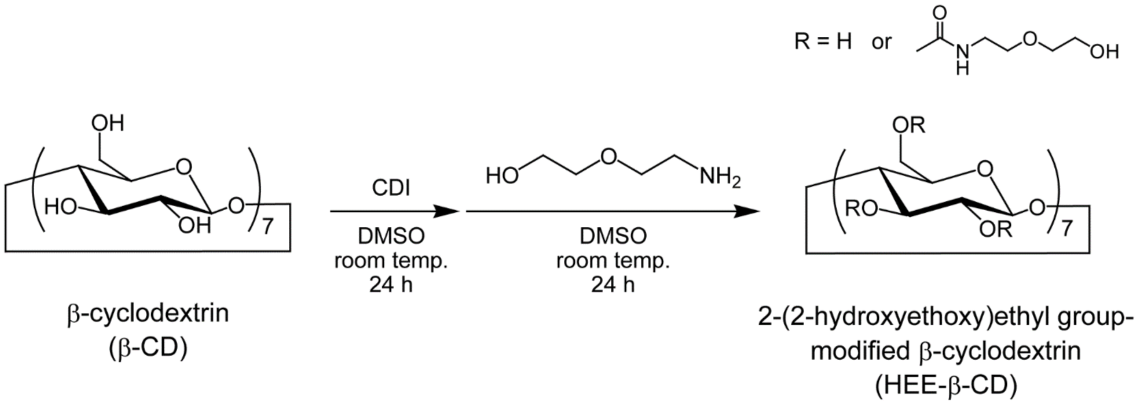

Figure S1. Reaction scheme for the synthesis of 2-(2-hydroxyethoxy)ethyl (HEE) group-modified β-CD (HEE-β-CD).

Table S1. Reaction conditions and characterization of HEE-β-CDs.

Sample code [a)]	Feed [HEEA]/[CDI]/ [β-CD] molar ratio [b)]	Number of HEE groups on β-CD [c)]	Averaged molecular weight [d)]
2.3HEE-β-CD	40/4/1	2.3	1,440
4.5HEE-β-CD	100/10/1	4.5	1,730
6.9HEE-β-CD	200/20/1	6.9	2,040

[a)] Abbreviated as *X*HEE-β-CD, where *X* denotes the average number of HEE groups modified on β-CD. [b)] The feed molar ratio of [HEEA]/[CDI] was kept constant at 10. [c)] Determined by ^{1}H NMR in D_2O. [d)] Calculated based on the chemical composition of the HEE-β-CDs determined by ^{1}H NMR.

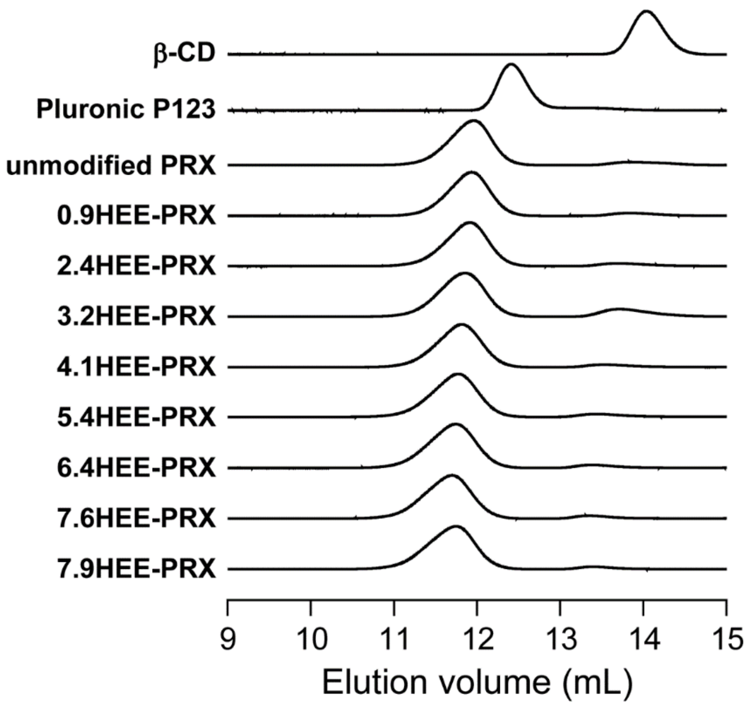

Figure S2. SEC charts of β-CD, Pluronic P123, unmodified PRX, and HEE-PRXs in DMSO containing 10 mM LiBr at 60 ºC.

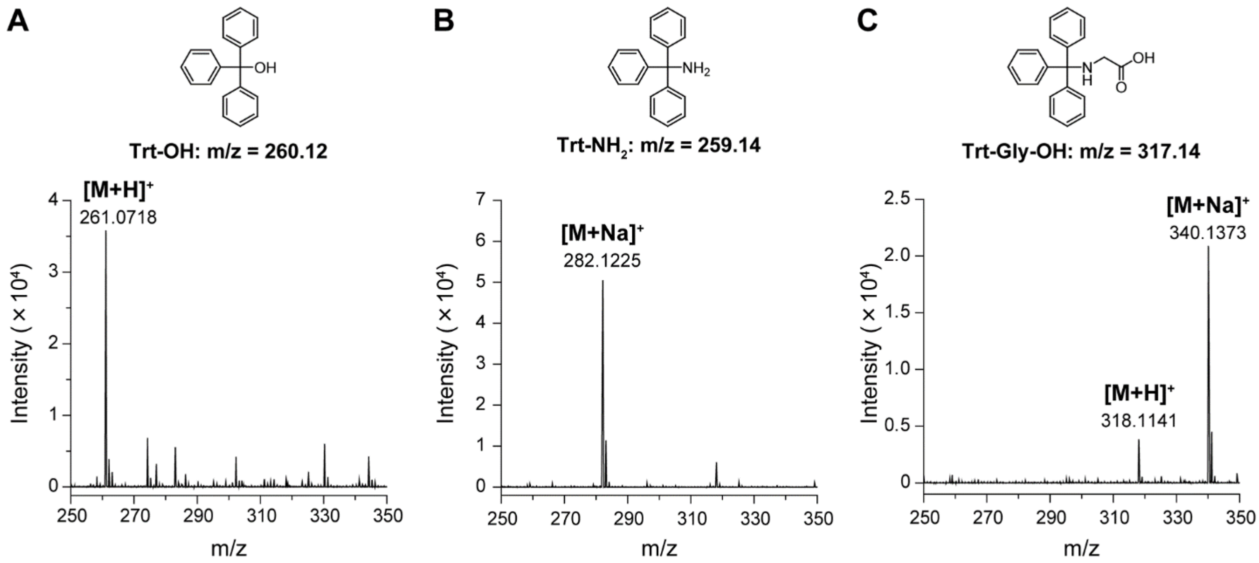

Figure S3. ESI-MS charts of Trt-OH (A), Trt-NH_2 (B), and Trt-Gly-OH (C).

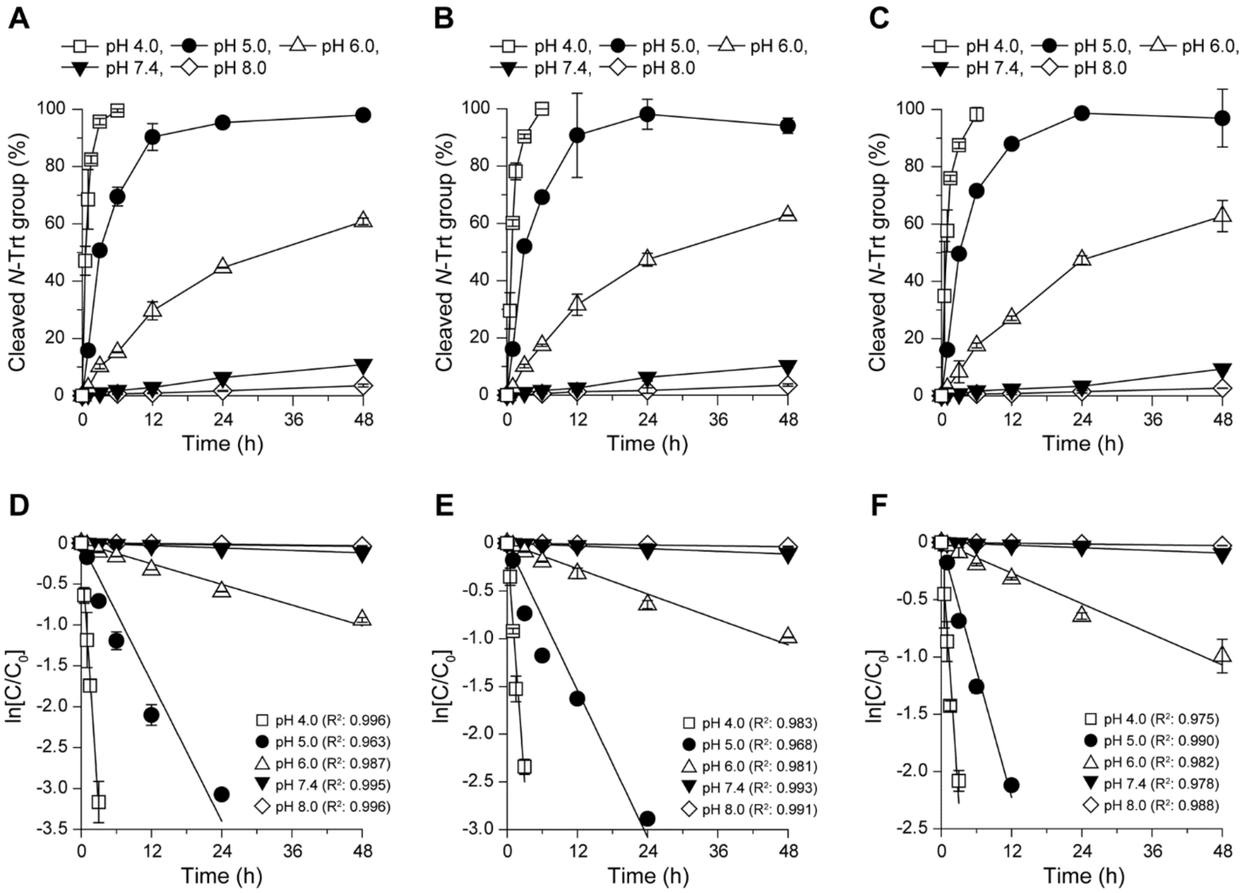

Figure S4. (A-C) Time course of the cleavage of *N*-Trt end groups in the HEE-PRXs (A: 5.4HEE-PRX, B: 6.4HEE-PRX, C: 7.6HEE-PRX) under various pH conditions (open squares: pH 4.0, closed circles: pH 5.0, open triangles: pH 6.0, closed triangles: pH 7.4, open diamonds: pH 8.0) at 37 ºC. (D-F) First-order kinetic plots for the cleavage of *N*-Trt end groups in the HEE-PRXs (D: 5.4HEE-PRX, E: 6.4HEE-PRX, F: 7.6HEE-PRX) under various pH conditions at 37 ºC. The data are expressed as the mean ± standard deviation (n = 3).

Table S2. First-order rate constants for the cleavage of the *N*-Trt groups and half-life of HEE-PRXs under various pH conditions at 37 ºC.

Sample code	pH	k (h^{-1})	$t_{1/2}$ (h)
4.1HEE-PRX	4.0	$8.6 \pm 0.4 \times 10^{-1}$	0.8
	5.0	$1.0 \pm 0.1 \times 10^{-1}$	6.8
	6.0	$1.5 \pm 0.1 \times 10^{-2}$	46
	7.4	$1.2 \pm 0.1 \times 10^{-3}$	587
	8.0	$4.4 \pm 0.1 \times 10^{-4}$	1580
5.4HEE-PRX	4.0	1.1 ± 0.1	0.6
	5.0	$1.4 \pm 0.1 \times 10^{-1}$	4.9
	6.0	$2.1 \pm 0.1 \times 10^{-2}$	33
	7.4	$2.5 \pm 0.1 \times 10^{-3}$	280
	8.0	$7.5 \pm 0.1 \times 10^{-4}$	930
6.4HEE-PRX	4.0	$8.3 \pm 0.6 \times 10^{-1}$	0.8
	5.0	$1.3 \pm 0.1 \times 10^{-1}$	5.4
	6.0	$2.2 \pm 0.1 \times 10^{-2}$	31
	7.4	$2.4 \pm 0.1 \times 10^{-3}$	295
	8.0	$7.5 \pm 0.4 \times 10^{-4}$	924
7.6HEE-PRX	4.0	$7.6 \pm 0.6 \times 10^{-1}$	0.9
	5.0	$1.9 \pm 0.1 \times 10^{-1}$	3.7
	6.0	$2.2 \pm 0.1 \times 10^{-2}$	31
	7.4	$1.9 \pm 0.1 \times 10^{-3}$	358
	8.0	$5.9 \pm 0.3 \times 10^{-4}$	1189

Table S3. First-order rate constants for the cleavage of the *N*-Trt groups and half-life of HEE-PRXs (4.1HEE-PRX) at various temperatures.

Sample code	pH	Temperature (ºC)	k (h^{-1})	$t_{1/2}$ (h)
4.1HEE-PRX	5.0	50	$1.6 \pm 0.1 \times 10^{-1}$	4.2
	5.0	37	$9.4 \pm 0.9 \times 10^{-2}$	7.4
	5.0	25	$4.5 \pm 0.6 \times 10^{-2}$	15
	5.0	4	$9.1 \pm 0.5 \times 10^{-3}$	76
	7.4	50	$3.4 \pm 0.1 \times 10^{-3}$	205
	7.4	37	$1.4 \pm 0.1 \times 10^{-3}$	492
	7.4	25	$1.0 \pm 0.1 \times 10^{-3}$	686
	7.4	4	$2.1 \pm 0.5 \times 10^{-4}$	3301

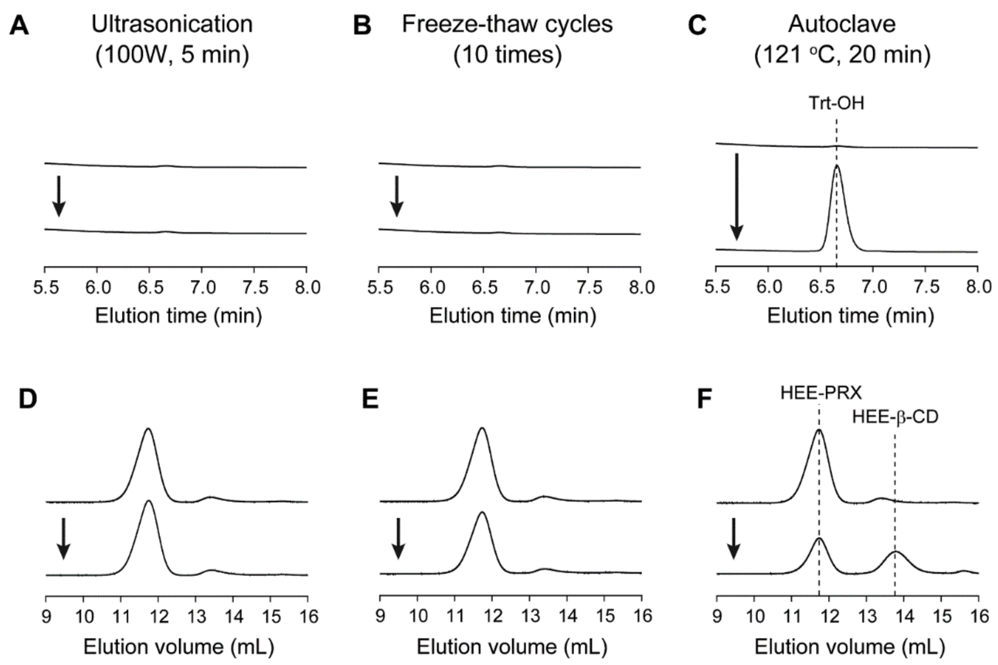

Figure S5. HPLC (A-C) and SEC charts (D-F) of 4.1HEE-PRX before (upper lines) and after the various treatments (lower lines); ultrasonication at 100 W for 5 min (A, D), freeze-thaw cycles 10 times (B, E), and autoclave at 121 ºC for 20 min (C, F).

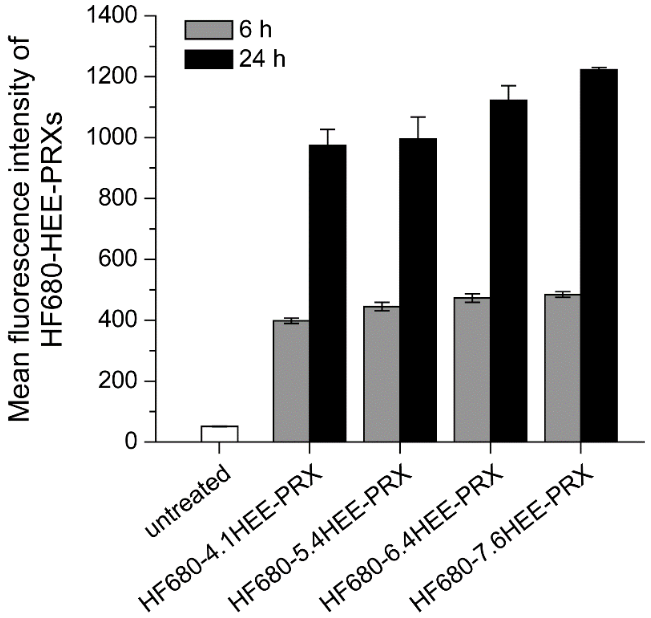

Figure S6. Mean fluorescence intensities of NPC1 fibroblasts treated with HF680-4.1HEE-PRXs (0.5 mM of β-CD) for 6 and 24 h (n = 3).

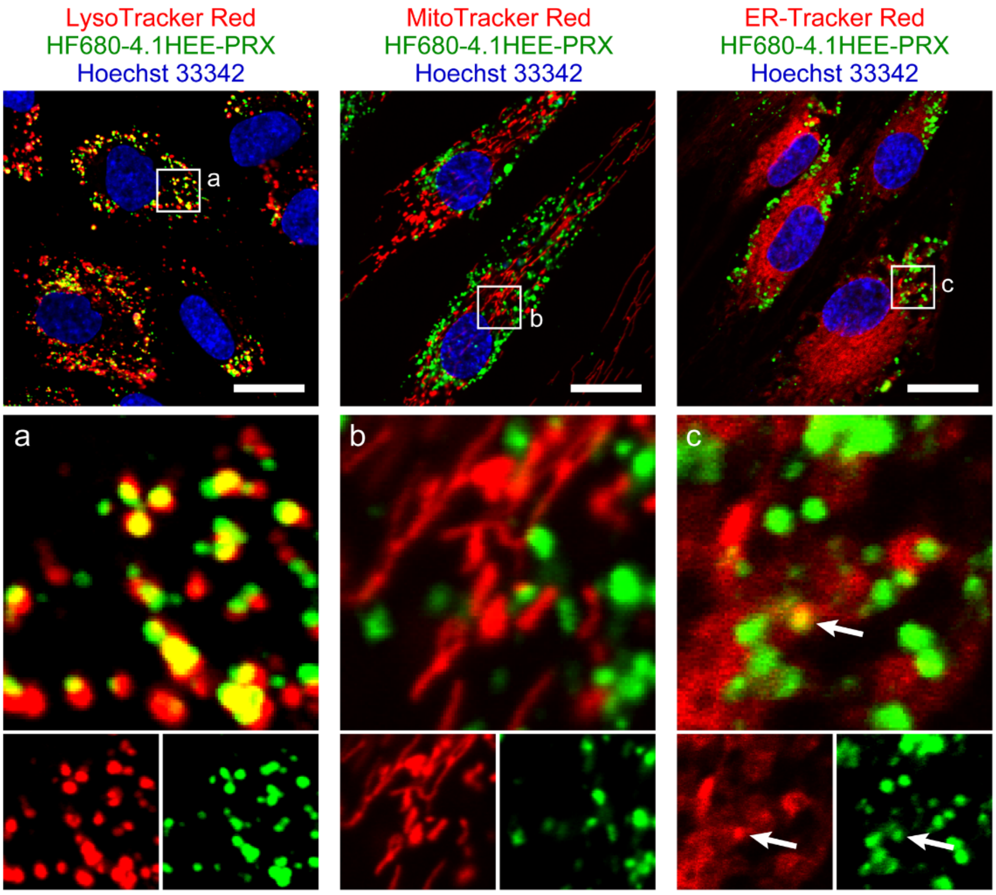

Figure S7. CLSM images of NPC1 fibroblasts treated with HF680-4.1HEE-PRX (0.5 mM of β-CD) for 24 h (scale bars: 20 μm). The endosomes/lysosomes, mitochondria, endoplasmic reticulum, and nuclei were stained with LysoTracker Red DND-99, MitoTracker Red CMXRos, ER-Tracker Red, and Hoechst 33342, respectively. Fluorescence colors are indicated by the color of the text. The bottom panels in each column show an enlarged view of the boxed regions. Colocalization of HF680-4.1HEE-PRX with each organelle is indicated by arrows.

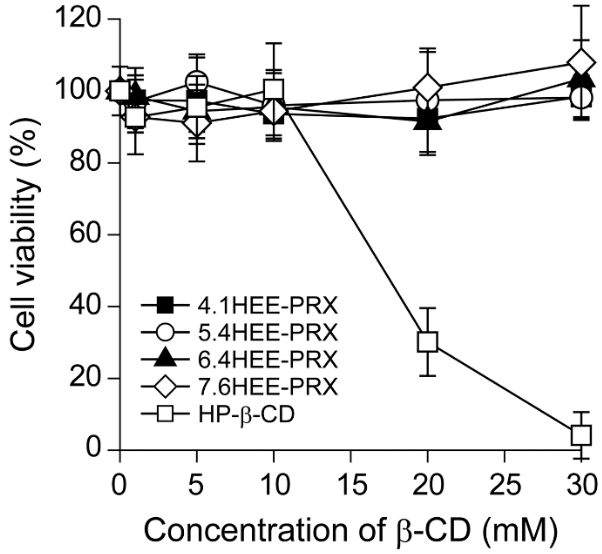

Figure S8. The viability of normal human skin fibroblasts treated with HEE-PRXs (4.1HEE-PRX: closed squares, 5.4HEE-PRX: open circles, 6.4HEE-PRX: closed triangles, 7.6HEE-PRX: open diamonds) and HP-β-CD (open squares) for 24 h (n = 4).
